# Mitochondrial disease patient motivations and barriers to participate in clinical trials

**DOI:** 10.1371/journal.pone.0197513

**Published:** 2018-05-17

**Authors:** Zarazuela Zolkipli-Cunningham, Rui Xiao, Amy Stoddart, Elizabeth M. McCormick, Amy Holberts, Natalie Burrill, Shana McCormack, Lauren Williams, Xiaoyan Wang, John L. P. Thompson, Marni J. Falk

**Affiliations:** 1 Division of Neurology, The Children’s Hospital of Philadelphia, Philadelphia, Pennsylvania, United States of America; 2 Mitochondrial Medicine Frontier Program, The Children’s Hospital of Philadelphia, Philadelphia, Pennsylvania, United States of America; 3 Department of Biostatistics and Epidemiology, University of Pennsylvania, Philadelphia, Pennsylvania, United States of America; 4 Arcadia University, Glenside, Pennsylvania, United States of America; 5 Division of Human Genetics, The Children’s Hospital of Philadelphia, Philadelphia, Pennsylvania, United States of America; 6 Rare Diseases Clinical Research Network, Health Informatics Institute, University of South Florida, Tampa, Florida, United States of America; 7 Division of Endocrinology and Diabetes, The Children’s Hospital of Philadelphia, Philadelphia, Pennsylvania, United States of America; 8 University of Pennsylvania Perelman School of Medicine, Philadelphia, Pennsylvania, United States of America; 9 Department of Biostatistics, Mailman School of Public Health, Columbia University Medical Center, New York, New York, United States of America; Texas Technical University Health Sciences Center, UNITED STATES

## Abstract

**Background:**

Clinical treatment trials are increasingly being designed in primary mitochondrial disease (PMD), a phenotypically and genetically heterogeneous collection of inherited multi- system energy deficiency disorders that lack effective therapy. We sought to identify motivating factors and barriers to clinical trial participation in PMD.

**Methods:**

A survey study was conducted in two independent mitochondrial disease subject cohorts. A discovery cohort invited subjects with well-defined biochemical or molecularly- confirmed PMD followed at a single medical center (CHOP, n = 30/67 (45%) respondents). A replication cohort included self-identified PMD subjects in the Rare Disease Clinical Research Network (RDCRN) national contact registry (n = 290/1119 (26%) respondents). Five-point Likert scale responses were analyzed using descriptive and quantitative statistics. Experienced and prioritized symptoms for trial participation, and patient attitudes toward detailed aspects of clinical trial drug features and study design.

**Results:**

PMD subjects experienced an average of 16 symptoms. Muscle weakness, chronic fatigue, and exercise intolerance were the lead symptoms encouraging trial participation.

Motivating trial design factors included a self-administered study drug; vitamin, antioxidant, natural or plant-derivative; pills; daily treatment; guaranteed treatment access during and after study; short travel distances; and late-stage (phase 3) participation. Relative trial participation barriers included a new study drug; discontinuation of current medications; disease progression; daily phlebotomy; and requiring participant payment. Treatment trial type or design preferences were not influenced by population age (pediatric versus adult), prior research trial experience, or disease severity.

**Conclusions:**

These data are the first to convey clear PMD subject preferences and priorities to enable improved clinical treatment trial design that cuts across the complex diversity of disease. Partnering with rare disease patient communities is essential to effectively design robust clinical trials that engage patients and enable meaningful evaluation of emerging treatment interventions.

## Introduction

Dramatic advances in genomic sequencing technologies have enabled marked improvements in the diagnosis and mechanistic understanding of primary mitochondrial disease (PMD). PMD is a highly heterogeneous collection of energy deficiency disorders, which are now recognized to result from more than 250 different gene disorders that can originate in either the nuclear DNA or mitochondrial DNA genome [1]. PMD are characterized by extensive clinical variability, with often progressive organ dysfunction occurring in nearly any system at any age. Collectively, PMD comprise the most common inborn error of metabolism but remain a rare disease, with minimal prevalence estimated at 1 in 4,300 individuals [2]. No FDA- approved treatments exist for mitochondrial disease, despite the first clinical trial for mitochondrial disease having been conducted in 1990 [3]. Two decades later, a systematic meta- analysis identified only 12 methodologically robust clinical trials, which yielded no efficacious evidence [4]. Hence, the vast majority of treatments used in mitochondrial disease patients are given on an empiric basis without having been objectively evaluated in robust clinical trials [5–9]. Current clinical practice for treating mitochondrial disease patients is therefore largely based upon clinical experience, small open label studies, case reports, and anecdotal evidence [5, 10, 11].

Rare disorders, such as PMD, are well-recognized to impose a distinct set of challenges in the classic clinical research infrastructure that do not easily fit into trial designs for common complex diseases [12–17]. Specific to PMD, each molecular subtype typically has very low prevalence with an increasing array of causal etiologies recognized, leading to a nearly four-fold increase in the number of disease-causing genes known to cause PMD over the last decade [1, 18, 19]. Given this inherent disease heterogeneity and rapidly changing diagnostic capabilities, their mechanistic basis and potential treatment approaches are only now being effectively deciphered [20]. Natural histories of many PMD subtypes has been lacking, although is increasingly prioritized for systematic study, such as through collaborative multi-site national initiatives including the NIH Rare Disease Clinical Research Network (RDCRN) in the North American Mitochondrial Disease Consortium (NAMDC, https://www.rarediseasesnetwork.org/cms/NAMDC). Establishment of multi-site national disease registries can further facilitate multi-center trial networks, which are ultimately necessary to enroll sufficient subjects with a given disease etiology or subtype in rare disease [21, 22].

However, the inherent clinical complexity of multi-system disorders such as PMD pose a substantial challenge to ready identification and prioritization of appropriate clinical trial end- point(s) and validated outcome measures [23–28]. Trial design characteristics have been challenging to standardize in PMD, including standard-of-care treatment regimen determination, study duration period, placebo-control, blinding, and randomization. Additional trial design challenges previously reported in in PMD have included subject recruitment and retainment [6], subject hesitation to discontinue current medications and dietary supplements [8, 28], travel barrier to study sites [6] and fear of potential adverse consequences [6, 9]. As such, many of the trials that have been designed in the past were based on investigator and/or pharmaceutical sponsor prioritized outcomes and trial design characteristics without widespread upfront engagement of the PMD patient perspective to inform them.

The nexus of improving PMD etiology and mechanistic understanding, appreciation for alternative trial designs for rare disease [29, 30] and a growing cadre of pharmaceutical research in new therapeutic targets for mitochondrial disease have brought renewed focus on the emerging potential to realize effective therapies for mitochondrial disease [28]. However, the success of this effort is contingent on successfully identifying PMD subject motivations and barriers to participate in clinical treatment trials. Here, we report results of an electronic survey designed to explore the patient perspective on these complex issues in exploratory and validation PMD cohorts, with substantial insights evident to improve clinical trial design in PMD.

## Methods

### Study design and PMD cohorts description

We designed and conducted an online electronic survey to determine the motivations and barriers to participate in clinical trials in adult and pediatric PMD patients, which consisted of questions divided into five key domains: Demographics, Symptom checklist of 35 options, Drug therapy, Study Design, and Additional Study Design Factors (S1 File). A few questions were developed to assess the validity of the responses. For analysis of patient attitudes toward clinical trial study design, subjects were asked if they would participate if half of the people receive placebo and the other half receive active drug, and asked separately if they would participate if half of the people get the active drug while the other half receive placebo. Given this is the same question, all subjects did respond identically for both questions. The initial survey was administered January through March 2014 in Research Electronic Data Capture (REDCap) to 67 invited subjects and families with definite or suspected mitochondrial disease enrolled in a Children’s Hospital of Philadelphia (CHOP) institutional review board approved study #08–6177 (MJF, PI) after providing written consent. Informed consent was obtained from parents/guardians of subjects <18 years of age. PMD subjects were defined as ‘definite’ when their molecular etiology was confirmed, and ‘suspected’ based only upon biochemical and/or clinical evidence. All returned surveys were anonymous to the study team, with diagnostic confirmation possible through cross-referencing gender, age, and zip code with the study database. Parents were asked to complete the survey on behalf of affected children, with a separate survey response completed on their own behalf if the parent themselves were also affected with PMD. The same online survey instrument was administered June through November 2014 via the RDCRN Data Management and Coordinating Center (DMCC) Oracle Database to a validation cohort of self-identified PMD subjects, and administered following a CHOP IRB waiver and RDCRN approval with mass-cohort email blast to 1,119 PMD subjects on two occasions. All ages were eligible to participate, where primary caregivers were requested to complete the survey for dependents. Inclusion criteria included definite or suspected mitochondrial disease, fluency in English and internet access. Incomplete surveys were excluded from analysis. Survey questions were closed-ended with responses along a 5-point Likert scale. Survey data was captured in REDCap for the initial CHOP-based PMD survey and in the Oracle Database for the validation RDCRN PMD survey.

### Statistical analyses

Affirmative responses were interpreted along a 5-point Likert scale of severity ranging from ‘most’ to ‘mild’ in the symptoms domain, and as ‘likely to participate’ and ‘would participate’ in all other categories. Results were prepared as tabulated descriptive statistics and presented as numbers (n) and percentage (%) of total respondents per question. A cohort-wide willingness to participate threshold ≥ 80% was considered to be clinically significant for evaluation of an individual item. Subgroup analysis of participants who completed the survey on behalf of themselves (≥18 years, adult), and participants who completed the survey on behalf of affected dependents (child) was not performed in the CHOP cohort due to small sample size, but was completed in the RDCRN validation survey (total respondents, n = 169 adults and n = 121 children). Chi-squared test was used to compare responses in the subgroup analysis. Logistic regression analysis was used to test the effect of having past experience in clinical research participation (n = 124) on the willingness to participate in a clinical trial. The Wilcoxon rank-sum test was used to analyze the association between subject PMD disease severity and permissible treatment burden (n = 263). All statistical analyses were performed with SAS 6.1 or higher (SAS Institute Inc, Cary, NC). P value < 0.05 was claimed to be statistically significant.

## Results

### Demographic factors and symptoms

**Fig 1:**
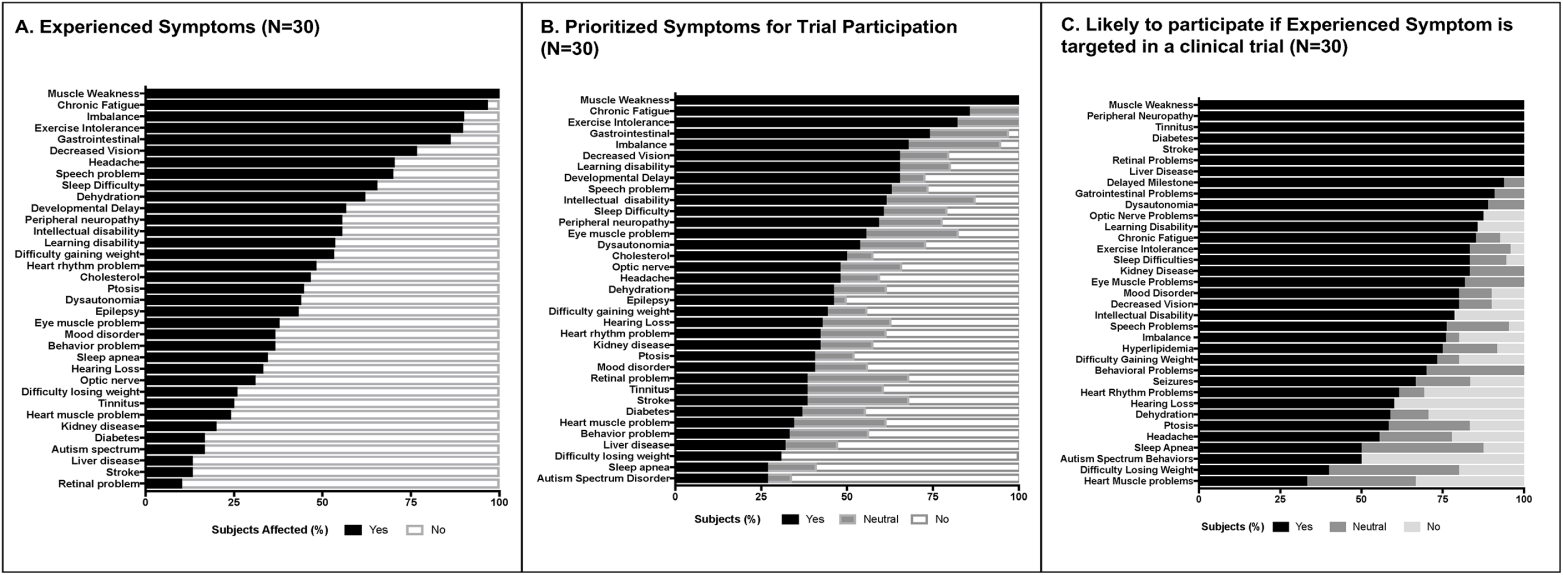
CHOP mitochondrial disease subject discovery cohort. **A. Experienced Symptoms.** The CHOP PMD subject cohort reported muscle weakness, chronic fatigue, exercise intolerance, imbalance, and gastrointestinal problems as the top 5 most commonly experienced symptoms (n = 30). **B. Prioritized Symptoms for trial participation.** The CHOP PMD patient cohort reported the top 5 most commonly experienced symptoms as the same leading symptoms to motivate their trial participation (n = 30). **C. Likely to participate if experienced symptom is targeted in a clinical trial.** All CHOP PMD subjects who experienced muscle weakness (n = 37), peripheral neuropathy (n = 13), tinnitus (n = 7), diabetes (n = 4), and stroke (n = 3) reported they would participate in a clinical trial that targeted these symptoms.

In the CHOP-based exploratory PMD cohort survey, a 45% respondent rate was obtained (30 of 67 invited participants with definite or suspected PMD). Demographic details are summarized in Table 1, with equal numbers of pediatric (n = 15) and adult (n = 15) PMD subject respondents. All subjects had definite or suspected PMD. Among the 35 symptom option selections, CHOP PMD subjects (n = 30) reported 16.3 ± 5.6 (mean ±SD, range 6–28) symptoms. Fig 1A and **Part A of** S1 Table detail the most commonly experienced symptoms, where the top 5 include: (1) muscle weakness, (2) chronic fatigue, (3) exercise intolerance; (4) imbalance, and (5) gastrointestinal problems. Specifically within the pediatric PMD subject cohort, however, developmental delay replaced gastrointestinal problems in the top 5 most common symptoms. When asked which symptom(s) would encourage trial participation, the same 5 prioritized symptoms were listed, including developmental delay in children (Fig 1B and **Part B of** S1 Table). When asked to select the top 3 symptoms each subject would prioritize for trial participation, muscle weakness, chronic fatigue, and exercise intolerance were ranked among the highest across the cohort. In the pediatric- specific subset, however, the top 3 prioritized symptoms for clinical trial participation included developmental delay, muscle weakness, and epilepsy (**Part D of** S1 Table). Targeted analysis of willingness to participate in a clinical trial only among those respondents who experience that particular symptom is shown in Fig 1C and **Part C of** S1 Table. Notably, this analysis revealed that all PMD subjects who have experienced muscle weakness (n = 27), peripheral neuropathy (n = 13), tinnitus (n = 7), diabetes (n = 4), and stroke (n = 3) would participate in a clinical trial that targeted these symptoms. Additionally, in the child group, all subjects with developmental delay (n = 14), learning disability (n = 10) and dysautonomia (n = 4) would participate in a trial.

**Table 1:** Demographic and other key characteristics of the CHOP patient cohort (N = 30).

	Adults (n = 15)	Children (n = 15)	p-value^1^
**Gender, Female—**% (n)	46.7 (7)	53.3 (8)	1.0
**Age, years: median (IQR)**	22–73 (46)	0.5–17 (7)	<.0001
**Diagnostic certainty–**% (n)			1.0
Definite	46.6 (7)	26.6 (4)	
Suspected	53.3 (8)	73.3 (11)	
	**Adults and Children (n = 30)**		
**Highest Education**	**% (n)**		
Less than high school degree	0 (0)		
High school degree or equivalent	13 (4)		
Some college but no degree	27 (8)		
Associate degree	3 (1)		
Bachelor degree	17 (5)		
Graduate degree	40 (12)		
**Family History**			
Yes	31.0 (9)		
**Previous Experience**			
Have participated in a previous research study	69 (20)		
Have participated in a clinical trial	34 (10)		
Know someone who participated in a clinical trial	41 (12)		

^1^Comparison by two-sample t-test between Adult and Child groups is indicated by p-value

**Fig 2:**
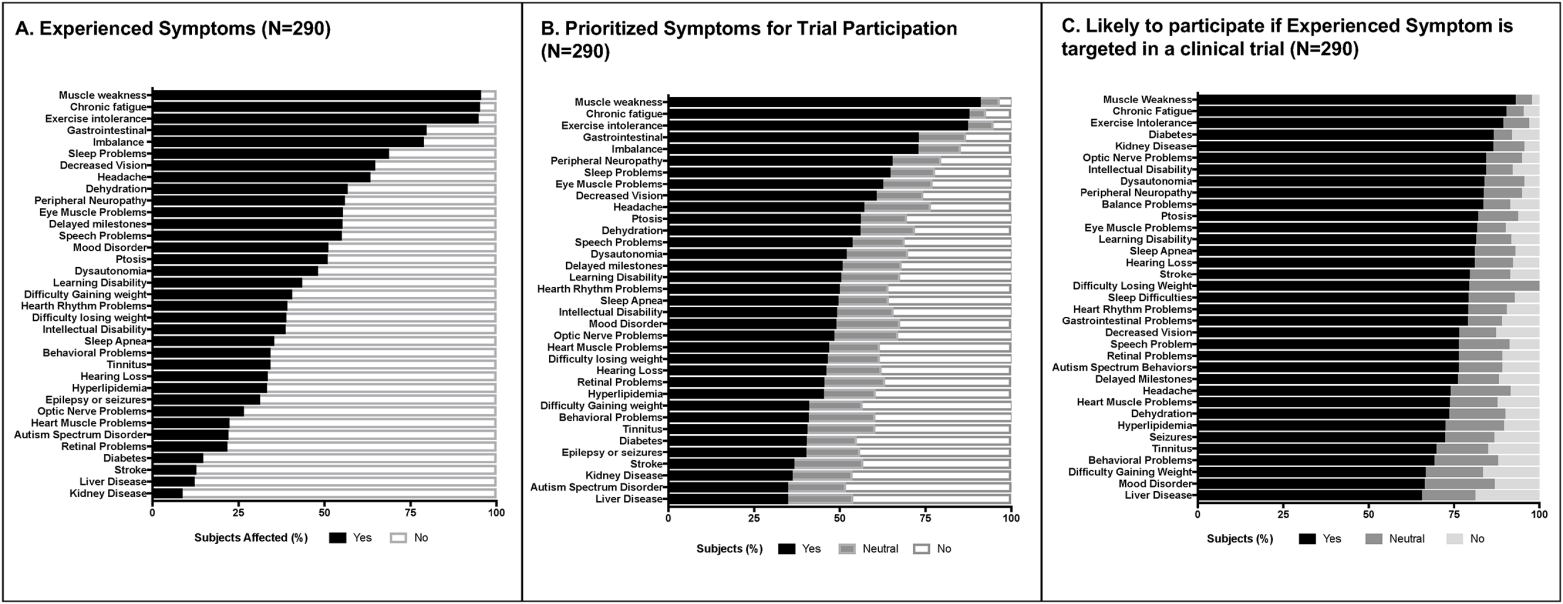
RDCRN mitochondrial disease subject validation cohort. **A. Experienced Symptoms.** Frequency of experienced symptoms as reported by the RDCRN self-reported cohort revealed muscle weakness, chronic fatigue, exercise intolerance, imbalance, and gastrointestinal problems to be the top 5 common symptoms (n = 290). **B. Prioritized Symptoms for trial participation.** The RDCRN cohort reported the same top 5 symptoms that would motivate trial participation (n = 290) as seen in the CHOP cohort (Fig 1). **C. Likely to participate if experienced symptom is targeted in a clinical trial.** The RDCRN cohort reported muscle weakness, chronic fatigue, exercise intolerance, diabetes and kidney disease as the most common experienced and desirable to treat symptoms in a clinical trial (n = 290).

In the RDCRN survey PMD validation cohort, a 26% response rate was obtained (298 of 1,119 RDCRN-enrolled invited participants). Eight responses were incomplete and excluded from analysis. Demographic details are summarized in Table 2, with n = 169 adults and n = 121 parents completing the survey for their children among the PMD subject respondents. In this self-reported PMD cohort, 40.7% of subjects reported having a definite molecular diagnosis, of whom 37.2% reported having mitochondrial DNA mutations. Identical to what was reported in the CHOP PMD cohort, RDCRN cohort subjects reported 15.6 ± 5.9 (mean ±SD, range 2–30) symptoms. The 5 most commonly experienced and 5 most prioritized symptoms for clinical trial participation were also identical to those identified in the CHOP PMD cohort survey (Fig 2A–2C **and Parts A-D of** S2 Table). Interestingly among the affirmative responses, ‘exercise intolerance’ was identified among the RDCRN cohort as the leading symptom in the ‘most severe’ category in all subjects and in the adult group (n = 169), as well as the second leading symptom after developmental delay in the pediatric group (n = 121) (S3 Table). The top 3 experienced symptoms that would encourage trial participation in adults (n = 169) were the same symptoms seen in the CHOP PMD cohort of chronic fatigue, muscle weakness and exercise intolerance although in the pediatric cohort (n = 121) exercise intolerance was replaced with gastrointestinal problems (n = 121, **Part D of** S2 Table). All RDCRN PMD cohort adults with kidney disease (n = 16) and all RDCRN PMD cohort children with diabetes (n = 4) expressed willingness to participate in a clinical trial (**Part C of** S2 Table).

**Table 2:** Demographic and other key characteristics of the RDCRN cohort (N = 290).

	Adults (n = 169)	Children (n = 121)	p-value^2^
**Female gender—**% (n)^1^	75.2 (124)	47.0 (52)	<.0001
**Age, years: median** (IQR)	48 (35–57)	12 (6–17)	<.0001
**Highest Education–**% (n)			
High school	12.5 (21)	-	
Some college	22.5 (38)	-	
College degree or equivalent	42.7 (72)	-	
Graduate degree	21.9 (37)	-	
No answer	0.6 (1)	-	
**Known mutation–**% (n)			0.4044
Yes	39.1 (66)	43.0 (52)	
No	21.3 (36)	25.6 (31)	
Maybe	38.5 (65)	31.4 (38)	
No answer	1.2 (2)	0 (0)	
**Mutation–**% (n)			0.0003
mtDNA	40.2 (68)	33.1 (40)	
Nuclear DNA	4.7 (8)	20.7 (25)	
Don’t know	49.1 (83)	43.8 (53)	
No answer	5.9 (10)	2.5 (3)	
**Family History–**% (n)	27.8 (47)	25.6 (31)	0.6782
**Previous Experience** % (n)			
Participated in research study	30.2 (51)	34.7 (42)	0.3416
Participated in clinical trial	8.9 (15)	13.2 (16)	0.4618
Acquaintance participated in clinical trial	16.0 (27)	33.9 (41)	0.0018

^1^Cases with missing data are excluded for gender (4 adults and 10 children), and Previous Experience (maximum 4). No data are missing on other variables in the table.

^2^Comparison by two-sample t-test between Adult and Child groups is indicated by p-value.

### Study drug characteristics

In the CHOP PMD cohort survey, subjects (n = 30) preferred if the study drug was a vitamin (96.7%), natural supplement (96.7%), food (93.3%), antioxidant (90.0%) or a plant product (83.3%) (S4 Table). Patients were less motivated to participate in a clinical trial if the study drug had been used for other diseases but not in PMD (70.0%). Trials requiring subjects to stop all of their medications (20.0%) or take a new drug (20.0%) were relative barriers to trial participation. Ingesting a pill (83.3%) as the route of administration, either once (93.1%) or twice (90.0%) a day, encouraged trial participation. An injection (46.7%), frequent (4 times daily) treatment administration (63.3%), nurse administered medication (43.3%), in-hospital administration (30.0%), and having an IV placed (48.1%) were discouraging factors to trial participation. Having the study drug (93.3%), a comparable drug (96.6%), or a new but unrelated drug (82.8%) made widely available to study participants after trial completion were encouraging factors. Experiencing symptom progression during the trial was a deterrent to trial continuation (53.3%). The RDCRN validation PMD cohort (n = 290) results showed the same pattern of motivating factors as were identified in the CHOP PMD cohort (n = 30) (S5 Table).

Subgroup analyses did not reveal significant differences.

### Study design features

In the CHOP cohort, subjects (n = 30) were motivated to participate if the study helped multiple (93.1%) or all (93.1%) symptoms, and the total study duration was no longer than one month (85.7%) (S4 Table). The prospect of receiving only placebo (33.3%), crossover (48.1%), double blind (29.6%) and randomized (37.0%) trial designs were relative barriers to trial participation, as was concurrent enrollment in another clinical trial (25.9%). Monthly blood tests (81.5%) were acceptable as were urine tests (80.8%), echocardiogram (85.2%), EKG (81.5%) and ultrasound (81.5%). Travel within a city was also acceptable (88.9%). While an offer of cash (55.6%) or gift card (55.6%) were not incentives, lack of monetary reimbursement (48.1%) was a deterrent.

The RDCRN survey revealed the same PMD subject preferences exist in the validation cohort (S5 Table). Additionally, acceptable designs included no requirement to travel (82.1%) or local travel (83.5%). Overnight stay (66.7%), domestic (61.0%) or international travel (39.7%) were less favored. Out-of-pocket medical expenses (13.3%) or costs to participate (18.9%) were major deterrents. Subjects were motivated to participate if the study was conducted by an academic hospital (86.2%) or local doctor (87.8%), as compared to a pharmaceutical company (64.2%). Participation in a later phase trial (Phase 3) was preferred, (81.2%) as compared to a phase 1 (58.9%) trial.

### Key study factors

Leading motivating factors and barriers incentives in the CHOP patient survey and RDCRN validation cohort are summarized in S4 and S5 Tables. Key motivating factors included the potential to cure (96.0%) and treat some disease symptoms (94.0%); as well as to prevent progression of disease (96.0%). The potential to benefit self (84.0%), family (84.0%), or other affected patients (80.0%) were more encouraging factors than to aid science (68.0%). The prospect of no existing alternative (84.0%) or affordable (84.0%) treatment options, or of no access to study drug outside of the trial (80.0%) motivated trial participation. Access to free healthcare (44.0%) was not an incentive, while potential out-of-pocket expenses (8.3%), potential to worsen disease state (8.3%), transient major side effects (8.3%) and death from study participation (0%) were profound deterrents. The potential for transient minor side effects was a discouraging factor (36.0%). Highlighting the importance of recruitment strategy, subjects were more likely to enroll if they learned of the trial through a medical specialist (88.0%), another trial participant (84.0%), a friend with mitochondrial disease (80.0%), phone call from the study team (80.0%) or email from the North American Mitochondrial Disease Consortium (NAMDC) (80.0%), as compared to from the general media. Genetic testing being performed in the study (80.0%) was not a deterrent, particularly if results did not affect their health insurance coverage (92.0%). This array of preferences was similarly reflected in the RDCRN validation cohort response (S5 Table**)**. Surprisingly however, 7% of subjects were willing to accept death as a potential risk of study participation.

### Past experience in a research study and motivation to participate

We hypothesized that subjects with past experience in a research study or clinical trial (n = 98) would remain motivated to participate in new treatment trials despite select study drug or design factors that had been reported by the overall survey group as unfavorable. However, logistic regression analysis of the RDCRN cohort data revealed that subjects with past research experience were not more willing to participate if the study drug had not been used in people before; if the medication was an injection; or if the study involved stopping one or all medications (S6 Table). Similarly, RDCRN cohort subjects were not more likely to participate if the study was more than 1 year in duration; involved daily blood tests; assigned a placebo- only arm; or involved double-blind or randomized design. Thus, prior study experience did not predict improved tolerance for generally unfavorable study design characteristics.

### Burden of disease and motivation to participate

We hypothesized that subjects with more severe disease would be willing to accept a higher burden of trial participation. Analysis of treatment burden was conducted by creating a total burden score of the i) Type of intervention (pill = 0, injection = 1); ii) Frequency of administration (below three = 0, three or more times a day = 1); and iii) Ease of administration (self-administrated = 0, administrated by nurse/hospital = 1) (n = 263). For each subject, the scores from each of these three dimensions were summed up to produce a total burden score and ranked. Results indicated no correlation existed between disease severity and level of treatment burden (S7 Table). Thus, higher disease severity did not predict acceptance of higher clinical trial participation burden.

## Discussion

The objective of this study was to identify motivating factors and barriers to clinical trial participation in mitochondrial disease, as is essential to inform clinicians, researchers, advocacy and regulatory partners, and pharmaceutical companies of PMD research subject needs and expectations for clinical trial participation. Results of the discovery survey in the well-defined CHOP PMD cohort were validated by analysis of a larger, self-identified PMD RDCRN cohort survey, confirming the consistency of specific needs and preferences across a wide array of subjects with PMD. Particularly striking was the discovery that subjects from both survey PMD cohorts reported experiencing a mean of 16 major clinical symptoms. This finding emphasizes the profound burden of PMD on patients, families, caregivers, and the health system [31]. In both survey cohorts we also identified the most prevalent and prioritized symptoms for clinical trial participation to be muscle weakness, exercise intolerance, fatigue, imbalance and gastrointestinal problems across all ages, as well as developmental delay in children.

Identifying reliable clinical outcomes and reliable measure(s) to assess their response to treatment in a clinical intervention trial represents the most fundamental steps in trial design. Learning the patient perspective of their most prevalent and disabling symptom(s) that they themselves prioritize for treatment is essential in PMDs that have such high phenotypic heterogeneity. This result is unique to PMD due to the multi-systemic nature of this disease, in contrast to studies in other rare inherited disorders [32, 33]. Awareness of discrepancies in physician-patient or physician-parent perceptions of disease burden need to be acknowledged and considered in trial design [34]. Patient-centered care demands understanding patient’s perspectives and working to meet their expectations [35, 36], which extends to clinical trials that aim to evaluate new treatment approaches. To design successful clinical trials, considering the patient rationale for clinical trial participation, expectations, and satisfaction will assure that mitochondrial disease research endeavors directly match PMD patient priorities and needs.

PMD patient disregard for study design elements that constitute a methodologically robust interventional clinical trial such as placebo control, blinding, and randomization was apparent in both surveys (S8 and S9 Tables and S1 Fig). While we postulated that PMD patients who had more severe disease burden or past experience of research study and/or clinical trial participation would be more amenable to accept these generally less favored trial design elements, study results did not confirm this (S6 and S7 Tables). Therefore, these survey results underscore the importance of educating PMD patients of the critical need to design and participate in methodologically robust trial, as less rigorous studies cannot reliably inform clinical decision making [5]. We propose widespread, coordinated efforts that involve PMD patient advocacy groups to organize community education sessions that clarify the components and need for efficacious clinical trial design.

Recruitment strategy was highlighted as a key variable in this study, where direct physician communication with PMD subjects was found most effective to motivate clinical trial participation. Recruitment for clinical trials in rare diseases can be aided by partnership with patient advocacy organizations and disease consortiums, particularly when a patient registry mechanism exists [16, 37]. The NIH RDCRN and United Mitochondrial Disease Foundation (UMDF) have patient-populated registries, respectively designed to recruit patients with all rare and specifically mitochondrial diseases. In addition, NAMDC has established a clinician- populated mitochondrial disease patient registry that provides the infrastructure to facilitate and expedite research collaboration and clinical trials. Indeed, strong partnership between advocacy organizations, researchers, patients, and families is a productive model that has been demonstrated to lead to treatment interventions for several rare diseases [16, 38, 39].

A common theme between our study and prior investigations in other rare diseases is the need to raise patient awareness of clinical trials, provide patient-centric solutions such as reducing travel time and costs, and engage patients in the study design [40–42]. Similarly, the duration and frequency of study visits, restrictions on concomitant drug use, and fear of clinical deterioration during trial participation have also been identified as common barriers in other rare diseases [40–42]. However, the inherent clinical complexity of PMD with highly variable multi- system findings gives rise to the central finding of this study, which is the need to understand the PMD patient perspective of incorporating their most prevalent and disabling symptom(s) into the clinical trial design. Thus, our report meaningfully extends the literature of patient motivation and barriers to participate in clinical trials.

A limitation of this study is the potential bias due to the response rate of 45% and 26% in the CHOP discovery and RDCRN validation survey cohort populations. PMD is a burdensome and highly morbid disease with frequent disease fluctuations triggered by acute stressors such as illness. In addition, more than one family member is often affected and parents of index cases may also be symptomatic. Indeed, the experience in our center with other surveys conducted in our complex and highly morbid clinical cohort has been a similar response rate of 30–45%. For these reasons, we consider the response rate of 46% attained in the CHOP clinic cohort to be satisfactory. The CHOP clinic cohort had an established relationship with the study investigators, which conceivably led to the higher response rate then the anonymized, national RDCRN cohort. Despite this, however, the CHOP cohort study results were closely replicated in the larger RDCRN cohort. Thus, it appears these 2 cohorts are representative of the broader PMD community. Recognizing relatively low response rate is a limiting factor, pursuing a larger, multi-center study of definite PMD patients to explore key findings from our study would likely further enhance our understanding of PMD patient motivations and barriers for trial participation.

In summary, this is the first study to report PMD patient preferences in terms of detailed motivations and barriers to their participation in clinical intervention trials. Incorporating patient prevalent symptoms, treatment needs, and trial expectations while improving education efforts to emphasize the need for conducting scientifically rigorous clinical trials are crucial factors in the development of clinically meaningful trials to develop efficacious therapies for PMD.

## Supporting information

S1 File Survey questionnaire. (PDF)

S1 Table **A. Symptoms Experienced by All patients, Adults and Children in the CHOP Discovery Cohort.** ^1^Nonrespondents on individual symptoms (maximum 3 [20%] for Adults and 3 [20%] for Children) are excluded. Lower denominator (N) indicates the total number of respondents. **B. All patients, Adults and Children likely to participate1 in a clinical trial, by symptom targeted.** ^1^Respondents coded “likely to participate” responded “Would Participate” or “Likely to participate” in a trial aiming to treat the listed symptom. ^2^Nonrespondents on individual symptoms (maximum of 4 [26.7]% for Adults and 2 for Children [13.3]%] are excluded. Lower denominator (N) indicates the total number of respondents. **C. All patients, Adults and Children likely to participate1 in a clinical trial if experienced symptom is targeted.** ^1^Respondents coded “likely to participate” responded “Would Participate” or “Likely to participate” in a trial aiming to treat the listed symptom. ^2^Nonrespondents on individual symptoms (maximum of 4 [26.7]% for Adults and for Children [13.3]%] are excluded. Lower denominator (N) indicates the total number of respondents. **D**. **Symptoms most frequently selected by individuals in the top 3**^**1**^ **that would prompt their participation in a clinical trial, among All patients, Adults and Children.** ^1^Respondents selected 3 symptoms from 35 symptoms listed. ^2^Nonrespondents on individual symptoms (maximum 2 [6.7%] for all patients are excluded. Lower denominator (N) indicates the total number of respondents.
(PDF)

S2 Table **A. Symptoms Experienced by All patients, Adults and Children.** ^1^Nonrespondents on individual symptoms (maximum for 16 [5.5%] Adults and 15 [5.2%] for Children) are excluded. Lower denominator (N) indicates the total number of respondents. **B. All patients, Adults and Children likely to participate1 in a clinical trial, by symptom targeted.** ^1^Respondents coded “likely to participate” responded “Would Participate” or “Likely to participate” in a trial aiming to treat the listed symptom. ^2^Nonrespondents on individual symptoms (maximum 27 [16.0%] for Adults and 14 [4.8%] for Children) are excluded. Lower denominator (N) indicates the total number of respondents. **C**. **All patients, Adults and Children likely to participate1 in a clinical trial if experienced symptom is targeted**. ^**1**^Respondents coded “likely to participate” responded “Would Participate” or “Likely to participate” in a trial aiming to treat the listed symptom. ^2^Nonrespondents on individual symptoms (maximum 258 [88.9%] for Adults and Children) are excluded. Lower denominator (N) indicates the total number of respondents. **D. Symptoms most frequently selected by individuals in the top 31 that would prompt their participation in a clinical trial, among All patients, Adults and Children.** ^1^Participants were asked to select, from a list of 35 symptoms, the top 3 that would prompt their participation in a clinical trial. ^2^Nonrespondents on individual symptoms (maximum 0 [0.0%] for Adults and 0 [0.0%] for Children) are excluded. Lower denominator (N) indicates the total number of respondents.
(PDF)

S3 Table Symptom severity in Adults and Children. ^1^Nonrespondents on individual symptoms are excluded.
(PDF)

S4 Table Likelihood to participate in clinical trials with differing design and other features in all patients (N = 30), Adults (N = 15) and Children (N = 15). (PDF)

S5 Table RDCRN survey (n = 290) respondents likely to participate in clinical trials with differing design and other features. ^1^Nonrespondents on individual symptoms (maximum n [33, 19.5%] for Adults and n [20, 16.5%] for Children) are excluded.
(PDF)

S6 Table Logistic regression analysis of impact of past research experience1 on preferences for study drug and design features among Adults and Children combined (N = 290). ^**1**^Respondents who had participated in a previous study or clinical trial were coded as having past research experience (N = 98).
(PDF)

S7 Table P-values from the Wilcoxon rank-sum test (n = 263) for severity score measurement. (PDF)

S8 Table Summary of likelihood of participation in a clinical trial in all CHOP(N = 30) and RDCRN (N = 290) subjects. (PDF)

S9 Table Summary of discouraging factors in a clinical trial among all CHOP (N = 30) and RDCRN (N = 290) subjects. (PDF)

S1 Fig Flow of PMD clinical trial based on patient perspectives and preferences. (TIFF)

## Abbreviations

CHOP: The Children’s Hospital of Philadelphia
IRB: Institutional Review Board
NAMDC: North American Mitochondrial Disease Consortium
PMD: Primary Mitochondrial Disease
RDRCN: Rare Diseases Clinical Research Network
REDCap: Research Electronic Data Capture
UMDF: United Mitochondrial Disease Foundation
