# Supplementary material for: Mitochondrial disease patient motivations and barriers to participate in clinical trials
Source: PLoS One. 2018 May 17;13(5):e0197513. doi: 10.1371/journal.pone.0197513 (PMC5957366; doi:10.1371/journal.pone.0197513)
Supplement: S1 File — (PDF) [file pone.0197513.s001.pdf]

PARTICIPANT

For whom are you completing this survey?

☐ Yourself
☐ Your child

DEMOGRAPHICS

What is your child's biological sex?
- ☐ Male
- ☐ Female

What is your child's age? ____________________________________

Does your child have a mitochondrial disease?
- ☐ Yes
- ☐ No

Does your child have a known genetic mutation that is clearly the cause of their mitochondrial disease?
- ☐ Yes
- ☐ No
- ☐ Don't know

Does your child's disease-causing mutation involve the nuclear DNA (nDNA) or mitochondrial DNA (mtDNA)?
- ☐ Nuclear DNA
- ☐ Mitochondrial DNA
- ☐ Don't know

What is your biological sex?
- ☐ Male
- ☐ Female

What is your age? ____________________________________

Do you have a mitochondrial disease?
- ☐ Yes
- ☐ No

Do you have a known genetic mutation that is clearly the cause of your mitochondrial disease?
- ☐ Yes
- ☐ No
- ☐ Don't know

Is the disease-causing mutation involving the nuclear DNA (nDNA) or mitochondrial DNA (mtDNA)?
- ☐ Nuclear DNA
- ☐ Mitochondrial DNA
- ☐ Don't know

What is the highest level of school you have completed or the highest degree you have received?
- ☐ Less than high school degree
- ☐ High school degree or equivalent (e.g., GED)
- ☐ Some college but no degree
- ☐ Associate degree
- ☐ Bachelor degree
- ☐ Graduate degree

What is your zip code? ____________________________________

Do you have a mitochondrial disease?
- ☐ Yes
- ☐ No

If you have children, do any of your children have a mitochondrial disease?
- ☐ Yes
- ☐ No
- ☐ Not applicable

FAMILY HISTORY

Please list all other family members with mitochondrial disease (e.g. parents, siblings, cousins). Type "none" if there is no additional family history of mitochondrial disease. __________________________________

PREVIOUS EXPERIENCE

Have you/your child ever participated in a previous research study?
- ☐ Yes
- ☐ No

Have you/your child ever participated in a clinical trial?
- ☐ Yes
- ☐ No

Do you know anyone that has participated in a clinical trial?
- ☐ Yes
- ☐ No

CURRENT SYMPTOMS

For questions that ask about you/your child, please respond based on who you are filling this survey out for (either for yourself or for your child).

Rate the severity of each of the following symptoms for you/your child.

	None or never	Mild	Moderate	Severe	Very severe
Chronic fatigue	☐	☐	☐	☐	☐
Exercise intolerance	☐	☐	☐	☐	☐
Muscle weakness	☐	☐	☐	☐	☐
Developmental delay	☐	☐	☐	☐	☐
Epilepsy or seizures	☐	☐	☐	☐	☐
Stroke	☐	☐	☐	☐	☐
Speech problem	☐	☐	☐	☐	☐
Behavioral problems	☐	☐	☐	☐	☐
Mood disorder	☐	☐	☐	☐	☐
Headache	☐	☐	☐	☐	☐
Intellectual disability	☐	☐	☐	☐	☐
Learning disability	☐	☐	☐	☐	☐
Autism-spectrum behaviors	☐	☐	☐	☐	☐
Dehydration	☐	☐	☐	☐	☐
Liver disease	☐	☐	☐	☐	☐
Kidney disease	☐	☐	☐	☐	☐
Diabetes	☐	☐	☐	☐	☐
High cholesterol and/or triglycerides	☐	☐	☐	☐	☐
Dysautonomia	☐	☐	☐	☐	☐
Hearing loss	☐	☐	☐	☐	☐
Tinnitus (ringing in ears)	☐	☐	☐	☐	☐
Decreased vision	☐	☐	☐	☐	☐
Retinal problems	☐	☐	☐	☐	☐
Optic nerve problems	☐	☐	☐	☐	☐
Eye muscle problems	☐	☐	☐	☐	☐
Ptosis (drooping eyelids)	☐	☐	☐	☐	☐
Balance problems	☐	☐	☐	☐	☐
Peripheral neuropathy	☐	☐	☐	☐	☐
Gastrointestinal problems	☐	☐	☐	☐	☐
Difficulty falling or staying asleep	☐	☐	☐	☐	☐
Sleep apnea	☐	☐	☐	☐	☐

Heart muscle problems	☐	☐	☐	☐	☐
Heart rhythm problems	☐	☐	☐	☐	☐
Difficulty gaining weight	☐	☐	☐	☐	☐
Difficulty losing weight	☐	☐	☐	☐	☐

Other symptoms (please specify) ____________________________________

DRUG THERAPY

Please rate how each item would affect your decisions to participate or not participate in a clinical trial.

For questions that ask about you/your child, please respond based on who you are filling this survey out for (either for yourself or for your child).

How likely would you/your child be to participate in a clinical trial if it involves...

	Would not Participate	Unlikely to Participate	Unsure	Likely to Participate	Would Participate
Taking a new drug that has never been used before on people	☐	☐	☐	☐	☐
Taking a drug that has been used for other purposes, but not for mitochondrial disease	☐	☐	☐	☐	☐
Taking a vitamin	☐	☐	☐	☐	☐
Taking an antioxidant	☐	☐	☐	☐	☐
Taking a natural supplement that is available at health food stores (ie. GNC)	☐	☐	☐	☐	☐
Taking a plant-derived product	☐	☐	☐	☐	☐
Taking a food product	☐	☐	☐	☐	☐
Participating in an exercise test	☐	☐	☐	☐	☐
Making no changes to your current medications	☐	☐	☐	☐	☐
Stopping one of your current medications	☐	☐	☐	☐	☐
Stopping all of your current medications	☐	☐	☐	☐	☐
Changing your diet	☐	☐	☐	☐	☐

DRUG THERAPY

Please rate how each item would affect your decisions to participate or not participate in a clinical trial.

For questions that ask about you/your child, please respond based on who you are filling this survey out for (either for yourself or for your child).

How likely would you/your child be to participate in a clinical trial if the drug...

	Would not Participate	Unlikely to Participate	Unsure	Likely to Participate	Would Participate
Is a pill	☐	☐	☐	☐	☐
Is an injection	☐	☐	☐	☐	☐
Has to be taken one time a day	☐	☐	☐	☐	☐
Has to be taken two times a day	☐	☐	☐	☐	☐
Has to be taken three times a day	☐	☐	☐	☐	☐
Has to be taken four or more times a day	☐	☐	☐	☐	☐
Can be self administered	☐	☐	☐	☐	☐
Has to be given by a nurse	☐	☐	☐	☐	☐
Has to be given at the hospital	☐	☐	☐	☐	☐

DRUG THERAPY

Please rate how each item would affect your decisions to participate or not participate in a clinical trial.

For questions that ask about you/your child, please respond based on who you are filling this survey out for (either for yourself or for your child).

If you/your child were enrolled in a clinical trial...

	Quit the clinical trial	Stay in the clinical trial
And the same drug became widely available to all people, would you...	☐	☐
And a comparable drug became widely available to all people, would you...	☐	☐
And a promising new but completely unrelated drug became widely available to all people, would you...	☐	☐
And you had progression of your disease symptoms while enrolled, would you...	☐	☐

GOAL OF THE STUDY

Please rate how each item would affect your decisions to participate or not participate in a clinical trial.

For questions that ask about you/your child, please respond based on who you are filling this survey out for (either for yourself or for your child).

How likely would you/your child be to participate in a clinical trial if it is supposed to help with the following ...

	Would not Participate	Unlikely to Participate	Unsure	Likely to Participate	Would Participate
Chronic fatigue	☐	☐	☐	☐	☐
Exercise intolerance	☐	☐	☐	☐	☐
Muscle weakness	☐	☐	☐	☐	☐
Developmental delay	☐	☐	☐	☐	☐
Epilepsy or seizures	☐	☐	☐	☐	☐
Stroke	☐	☐	☐	☐	☐
Speech problem	☐	☐	☐	☐	☐
Behavioral problems	☐	☐	☐	☐	☐
Mood disorder	☐	☐	☐	☐	☐
Headache	☐	☐	☐	☐	☐
Intellectual disability	☐	☐	☐	☐	☐
Learning disability	☐	☐	☐	☐	☐
Autism-spectrum behaviors	☐	☐	☐	☐	☐
Dehydration	☐	☐	☐	☐	☐
Liver disease	☐	☐	☐	☐	☐
Kidney disease	☐	☐	☐	☐	☐
Diabetes	☐	☐	☐	☐	☐
High cholesterol and/or triglycerides	☐	☐	☐	☐	☐
Dysautonomia	☐	☐	☐	☐	☐
Hearing loss	☐	☐	☐	☐	☐
Tinnitus (ringing in ears)	☐	☐	☐	☐	☐
Decreased vision	☐	☐	☐	☐	☐
Retinal problems	☐	☐	☐	☐	☐
Optic nerve problems	☐	☐	☐	☐	☐
Eye muscle problems	☐	☐	☐	☐	☐
Ptosis (drooping eyelids)	☐	☐	☐	☐	☐

Balance problems	☐	☐	☐	☐	☐
Peripheral neuropathy	☐	☐	☐	☐	☐
Gastrointestinal problems	☐	☐	☐	☐	☐
Difficulty falling or staying asleep	☐	☐	☐	☐	☐
Sleep apnea	☐	☐	☐	☐	☐
Heart muscle problems	☐	☐	☐	☐	☐
Heart rhythm problems	☐	☐	☐	☐	☐
Difficulty gaining weight	☐	☐	☐	☐	☐
Difficulty losing weight	☐	☐	☐	☐	☐

What are the TOP 3 symptoms that would prompt you/your child to participate in a clinical trial? (Please pick 3 and only 3)

☐ Chronic fatigue
☐ Exercise intolerance
☐ Muscle weakness
☐ Developmental delay
☐ Epilepsy or seizures
☐ Stroke
☐ Speech problem
☐ Behavioral problems
☐ Mood disorder
☐ Headache
☐ Intellectual disability
☐ Learning disability
☐ Autism-spectrum behaviors
☐ Dehydration
☐ Liver disease
☐ Kidney disease
☐ Diabetes
☐ High cholesterol and/or triglycerides
☐ Dysautonomia
☐ Hearing loss
☐ Tinnitus (ringing in ears)
☐ Decreased vision
☐ Retinal problems
☐ Optic nerve problems
☐ Eye muscle problems
☐ Ptosis (drooping eyelids)
☐ Balance problems
☐ Peripheral neuropathy
☐ Gastrointestinal problems
☐ Difficulty falling or staying asleep
☐ Sleep apnea
☐ Heart muscle problems
☐ Heart rhythm problems
☐ Difficulty gaining weight
☐ Difficulty losing weight

GOAL OF THE STUDY

Please rate how each item would affect your decisions to participate or not participate in a clinical trial.

For questions that ask about you/your child, please respond based on who you are filling this survey out for (either for yourself or for your child).

How likely would you/your child be to participate in a clinical trial if it is supposed to help with ...

	Would not Participate	Unlikely to Participate	Unsure	Likely to Participate	Would Participate
Only one of the symptoms that you/your child experiences from the mitochondral disease	☐	☐	☐	☐	☐
Multiple symptoms that you/your child experience but not all of the symptoms	☐	☐	☐	☐	☐
All of the symptoms you/your child experience from the mitochondrial disease	☐	☐	☐	☐	☐

DESIGN OF THE STUDY

Please rate how each item would affect your decisions to participate or not participate in a clinical trial.

For questions that ask about you/your child, please respond based on who you are filling this survey out for (either for yourself or for your child).

How likely would you/your child be to participate in a clinical trial if it is...

	Would not Participate	Unlikely to Participate	Neutral this would not affect the decision	Likely to Participate	Would Participate
One day long	☐	☐	☐	☐	☐
One week long	☐	☐	☐	☐	☐
One month long	☐	☐	☐	☐	☐
Several months (3-4 months) in length	☐	☐	☐	☐	☐
One year in length	☐	☐	☐	☐	☐
More than one year in length	☐	☐	☐	☐	☐

DESIGN OF THE STUDY

Please rate how each item would affect your decisions to participate or not participate in a clinical trial.

For questions that ask about you/your child, please respond based on who you are filling this survey out for (either for yourself or for your child).

How likely would you/your child be to participate in a clinical trial that is designed so that...

	Would not Participate	Unlikely to Participate	Neutral this would not affect the decision	Likely to Participate	Would Participate
Half of the people in the study get a placebo pill (inactive drug) and the other half get the active drug	☐	☐	☐	☐	☐
Half of the people in the study get the active drug and the other half get a placebo pill (inactive drug)	☐	☐	☐	☐	☐
You sequentially take several different drugs or placebos each for a defined time period in an unpredictable order (ie. take drug A for one month, then take drug B for one month, then take drug C for one month)	☐	☐	☐	☐	☐
There is a chance of only getting the placebo (inactive drug)	☐	☐	☐	☐	☐
Everyone gets the drug and placebo at some point	☐	☐	☐	☐	☐
Everyone gets the drug and no one gets a placebo	☐	☐	☐	☐	☐
Neither you nor the study team know whether you are receiving the drug or placebo	☐	☐	☐	☐	☐
Only you do not know which treament you are receiving	☐	☐	☐	☐	☐
Only your doctor does not know which treatment you are receiving	☐	☐	☐	☐	☐
The study team selects whether you receive the drug or placebo	☐	☐	☐	☐	☐
You select whether you receive the drug or placebo	☐	☐	☐	☐	☐

There is random assignment of who receives the drug or placebo	☐	☐	☐	☐	☐
You could be randomized to either take the new treatmeat or continue your regular mitochondrial cocktail	☐	☐	☐	☐	☐
You are already enrolled in another clinical trial at the same time	☐	☐	☐	☐	☐
You are guaranteed the drug after the study ends	☐	☐	☐	☐	☐

DESIGN OF THE STUDY

Please rate how each item would affect your decisions to participate or not participate in a clinical trial.

For questions that ask about you/your child, please respond based on who you are filling this survey out for (either for yourself or for your child).

How likely would you/your child be to participate if the clinical trial involves...

	Would not Participate	Unlikely to Participate	Neutral this would not affect the decision	Likely to Participate	Would Participate
Daily blood tests	☐	☐	☐	☐	☐
Weekly blood tests	☐	☐	☐	☐	☐
Monthly blood tests	☐	☐	☐	☐	☐
2 blood tests (one at the beginning and one at the end)	☐	☐	☐	☐	☐
Urine tests	☐	☐	☐	☐	☐
Stool tests	☐	☐	☐	☐	☐
An electrocardiogram (ECG)	☐	☐	☐	☐	☐
An echocardiogram (heart ultrasound)	☐	☐	☐	☐	☐
Heart rate monitoring	☐	☐	☐	☐	☐
Exercise tests	☐	☐	☐	☐	☐
An X-ray	☐	☐	☐	☐	☐
An MRI	☐	☐	☐	☐	☐
An ultrasound	☐	☐	☐	☐	☐
Having an IV placed	☐	☐	☐	☐	☐
Visits to the research site or a hospital	☐	☐	☐	☐	☐
Overnight hospital visits	☐	☐	☐	☐	☐
No traveling at all	☐	☐	☐	☐	☐
Traveling within your city or town	☐	☐	☐	☐	☐
Traveling to another state	☐	☐	☐	☐	☐
International travel to another country	☐	☐	☐	☐	☐
Traveling while you are experiencing symptoms	☐	☐	☐	☐	☐
Traveling when you are feeling good enough to travel	☐	☐	☐	☐	☐

No payment or monetary reimbursement	☐	☐	☐	☐	☐
A cash incentive to participate	☐	☐	☐	☐	☐
A gift card incentive to participate	☐	☐	☐	☐	☐
You having to make a payment in order to be part of the trial	☐	☐	☐	☐	☐

DESIGN OF THE STUDY

Please rate how each item would affect your decisions to participate or not participate in a clinical trial.

For questions that ask about you/your child, please respond based on who you are filling this survey out for (either for yourself or for your child).

How likely would you/your child be to participate if the clinical trial is ...

	Would not Participate	Unlikely to Participate	Neutral this would not affect the decision	Likely to Participate	Would Participate
Conducted by your local doctor	☐	☐	☐	☐	☐
Conducted by an academic hospital	☐	☐	☐	☐	☐
Conducted by a pharmaceutical company	☐	☐	☐	☐	☐
Conducted by a patient advocacy group or support group	☐	☐	☐	☐	☐
A single-site trial	☐	☐	☐	☐	☐
A multi-site trial (different locations are working together on the same trial)	☐	☐	☐	☐	☐
In phase 1 (screening for safety)	☐	☐	☐	☐	☐
In phase 2 (establishing the efficacy of the drug, usually against a placebo)	☐	☐	☐	☐	☐
In phase 3 (final confirmation of safety and efficacy)	☐	☐	☐	☐	☐

ADDITIONAL FACTORS

Please rate how each item would affect your decisions to participate or not participate in a clinical trial.

Would the following factor(s) influence your decision to participate in a clinical trial:

	Yes more likely to participate	Yes less likely to participate	No would not influence
Potential to benefit yourself	☐	☐	☐
Potential to benefit your family	☐	☐	☐
Potential to benefit other affected individuals	☐	☐	☐
Potential to aid in science and scientific advancement	☐	☐	☐
No other treatment options exist	☐	☐	☐
No other affordable treatment options	☐	☐	☐
The same treatment is not available clinically	☐	☐	☐
The same treatment is available outside of the trial but too expensive to access	☐	☐	☐
Access to free healthcare	☐	☐	☐
Apparent risks will outweigh the benefit	☐	☐	☐
Apparent benefits will outweigh the risks	☐	☐	☐
No prospective self benefit	☐	☐	☐
Possibility to cure your disease	☐	☐	☐
Possibility to prevent progression of your disease	☐	☐	☐
Possibility to treat some symptoms of your disease	☐	☐	☐
Potential of worsening your disease	☐	☐	☐
Potential of experiencing transient major side effects	☐	☐	☐
Potential of experiencing transient minor side effects	☐	☐	☐
Potential for death from study participation	☐	☐	☐

Potential for closer monitoring of your health	☐	☐	☐
Potential out-of-pocket expenses	☐	☐	☐
Desire to participate in any clinical trial	☐	☐	☐
Desire to avoid participation in any clinical trial	☐	☐	☐

ADDITIONAL FACTORS

Please rate how each item would affect your decisions to participate or not participate in a clinical trial.

For questions that ask about you/your child, please respond based on who you are filling this survey out for (either for yourself or for your child).

How likely would you/your child be to participate in a clinical trial if you learned about the trial through...

	Would not Participate	Unlikely to Participate	this would not affect the decision	Likely to Participate	Would Participate
Your primary care physician	☐	☐	☐	☐	☐
One of your medical specialists	☐	☐	☐	☐	☐
A healthy family member	☐	☐	☐	☐	☐
A family member that was already in the clinical trial	☐	☐	☐	☐	☐
A healthy friend	☐	☐	☐	☐	☐
A friend who also has a mitochondrial disease	☐	☐	☐	☐	☐
Another participant that was already in the clinical trial	☐	☐	☐	☐	☐
A support group or patient advocacy group	☐	☐	☐	☐	☐
The NIH clinical trials website	☐	☐	☐	☐	☐
A newspaper article	☐	☐	☐	☐	☐
A social media website	☐	☐	☐	☐	☐
The internet	☐	☐	☐	☐	☐
The television	☐	☐	☐	☐	☐
A flyer	☐	☐	☐	☐	☐
A letter mailed to your home	☐	☐	☐	☐	☐
A phonecall from the study team	☐	☐	☐	☐	☐
An email from the study team	☐	☐	☐	☐	☐
An email from the North American Mitochondrial Disease Consortium (NAMDC)	☐	☐	☐	☐	☐

ADDITIONAL FACTORS

Please rate how each item would affect your decisions to participate or not participate in a clinical trial.

For questions that ask about you/your child, please respond based on who you are filling this survey out for (either for yourself or for your child).

How likely are you/your child to participate in a clinical trial knowing that...

	Would not Participate	Unlikely to Participate	this would not affect the decision	Likely to Participate	Would Participate
Your genetic information is being analyzed	☐	☐	☐	☐	☐
Your genetic information cannot affect your medical insurance policy	☐	☐	☐	☐	☐
Your genetic information can affect your ability to purchase a life insurance policy	☐	☐	☐	☐	☐
Your genetic information can affect your ability to qualify for disability insurance	☐	☐	☐	☐	☐

COMMENTS

Please provide any additional comments: ________________________________

Do you plan to fill out the survey again for either yourself or your child?
- ☐ Yes
- ☐ No
