# Supplementary material for: Mitochondrial disease patient motivations and barriers to participate in clinical trials
Source: PLoS One. 2018 May 17;13(5):e0197513. doi: 10.1371/journal.pone.0197513 (PMC5957366; doi:10.1371/journal.pone.0197513)
Supplement: S1 Table — A. Symptoms Experienced by All patients, Adults and Children in the CHOP Discovery Cohort. 1Nonrespondents on individual symptoms (maximum 3 [20%] for Adults and 3 [20%] for Children) are excluded. Lower denominator (N) indicates the total number of respondents. B. All patients, Adults and Children likely to participate1 in a clinical trial, by symptom targeted. 1Respondents coded “likely to participate” responded “Would Participate” or “Likely to participate” in a trial aiming to treat the listed symptom. 2Nonrespondents on individual symptoms (maximum of 4 [26.7]% for Adults and 2 for Children [13.3]%] are excluded. Lower denominator (N) indicates the total number of respondents. C. All patients, Adults and Children likely to participate1 in a clinical trial if experienced symptom is targeted. 1Respondents coded “likely to participate” responded “Would Participate” or “Likely to participate” in a trial aiming to treat the listed symptom. 2Nonrespondents on individual symptoms (maximum of 4 [26.7]% for Adults and for Children [13.3]%] are excluded. Lower denominator (N) indicates the total number of respondents. D. Symptoms most frequently selected by individuals in the top 31 that would prompt their participation in a clinical trial, among All patients, Adults and Children. 1Respondents selected 3 symptoms from 35 symptoms listed. 2Nonrespondents on individual symptoms (maximum 2 [6.7%] for all patients are excluded. Lower denominator (N) indicates the total number of respondents. (PDF) [file pone.0197513.s002.pdf]

S1A Table. Symptoms Experienced by All patients, Adults and Children.

All (N=30)[1]		Adults (N=15)[1]		Children (N=15)[1]	
**Symptom**	**% (n)**	**Symptom**	**% (n)**	**Symptom**	**% (n)**
Muscle weakness	100.0 (30/30)	Muscle weakness	100.0 (15/15)	Muscle weakness	100.0 (15/15)
Chronic Fatigue	96.7 (29/30)	Chronic fatigue	100.0 (15/15)	Developmental Delay	100.0 (15/15)
Balance Problem	90.0 (27/30)	Gastrointestinal	93.3 (14/15)	Chronic Fatigue	93.3 (14/15)
Exercise Intolerance	89.7 (26/29)	Exercise Intolerance	92.9 (13/14)	Balance Problem	93.3 (14/15)
Gastrointestinal	86.2 (25/29)	Balance Problem	86.7 (13/15)	Exercise Intolerance	86.7 (13/15)
Decreased Vision	76.7 (23/30)	Decreased Vision	80.0 (12/15)	Speech problem	86.7 (13/15)
Headache	70.4 (19/27)	Difficulty sleeping	78.6 (11/14)	Intellectual Disability	84.6 (11/13)
Speech problem	70.0 (21/30)	Headache	73.3 (11/15)	Learning Disability	78.6 (11/14)
Difficulty Sleeping	65.5 (19/29)	Peripheral neuropathy	71.4 (10/14)	Gastrointestinal	78.6 (11/14)
Dehydration	62.1 (18/29)	Dehydration	66.7 (10/15)	Decreased Vision	73.3 (11/15)
Developmental Delay	56.7 (17/30)	Hypercholesteremia	66.7 (10/15)	Difficulty gaining weight	73.3 (11/15)
Intellectual Disability	55.6 (15/27)	Ptosis	64.3 (9/14)	Epilepsy	66.7 (10/15)
Peripheral Neuropathy	55.6 (15/27)	Speech problem	53.3 (8/15)	Headache	66.7 (8/12)
Learning Disability	53.6 (15/28)	Hearing Loss	53.3 (8/15)	Dehydration	57.1 (8/14)
Difficulty Gaining Weight	53.3 (16/30)	Dysautonomia	50.0 (6/12)	Difficulty Sleeping	53.3 (8/15)
Heart Rhythm problems	48.3 (14/29)	Heart Rhythm problems	50.0 (7/14)	Heart Rhythm problems	46.7 (7/15)
Hypercholesterolemia	46.7 (14/30)	Mood disorder	46.7 (7/15)	Behavior problems	40.0 (6/15)
Ptosis	44.8 (13/29)	Tinnitus	46.7 (7/15)	Dysautonomia	38.5 (5/13)
Dysautonomia	44.0 (11/25)	Eye muscle problems	42.9 (6/14)	Peripheral neuropathy	38.5 (5/13)
Epilepsy	43.3 (13/30)	Difficulty losing weight	38.5 (5/13)	Optic Nerve problems	35.7 (5/14)
Eye muscle problems	37.9 (11/29)	Behavioral Problems	33.3 (5/15)	Sleep Apnea	35.7 (5/14)
Behavioral problems	36.7 (11/30)	Diabetes	33.3 (5/15)	Eye muscle problems	33.3 (5/15)
Mood Disorder	36.7 (11/30)	Sleep apnea	33.3 (5/15)	Autism Spectrum	33.3 (5/15)

Sleep apnea	34.5 (10/29)	Difficulty gaining weight	33.3 (5/15)	Mood Disorder	26.7 (4/15)
Hearing Loss	33.3 (10/30)	Intellectual Disability	28.6 (4/14)	Kidney Disorders	26.7 (4/15)
Optic nerve problems	31.0 (9/29)	Learning Disability	28.6 (4/14)	Hypercholeste rolemia	26.7 (4/15)
Difficulty losing weight	25.9 (7/27)	Heart muscle problems	28.6 (4/14)	Ptosis	26.7 (4/15)
Tinnitus	25.0 (7/28)	Optic nerve problems	26.7 (4/15)	Heart Muscle problems	20.0 (3/15)
Heart muscle problems	24.1 (7/29)	Epilepsy	20.0 (3/15)	Retinal problems	14.3 (2/14)
Kidney Disorders	20.0 (6/30)	Liver disease	20.0 (3/15)	Difficulty losing weight	14.3 (2/14)
Autism Spectrum	16.7 (5/30)	Developmental Delay	13.3 (2/15)	Stroke	13.3 (2/15)
Diabetes	16.7 (5/30)	Stroke	13.3 (2/15)	Hearing Loss	13.3 (2/15)
Stroke	13.3 (4/30)	Kidney Disorders	13.3 (2/15)	Liver disease	6.7 (1/15)
Liver disease	13.3 (4/30)	Retinal problems	6.7 (1/15)	Diabetes	0 (0/15)
Retinal problems	10.3 (3/29)	Autism Spectrum	0.0 (0/15)	Tinnitus	0 (0/13)

[1]Nonrespondents on individual symptoms (maximum 3 [20%] for Adults and 3 [20%] for Children) are excluded. Lower denominator (N) indicates the total number of respondents.

S1B Table. All patients, Adults and Children likely to participate[1] in a clinical trial, by symptom targeted.

All (N=30)[2]		Adults (N=15)[2]		Children (N=15)[2]	
**Symptom targeted by the trial**	**% (n)**	**Symptom targeted by the trial**	**% (n)**	**Symptom targeted by the trial**	**% (n)**
Muscle weakness	100.0 (27/27)	Exercise intolerance	100.0 (14/14)	Muscle weakness	100.0 (13/13)
Chronic fatigue	85.7 (24/28)	Muscle weakness	100.0 (14/14)	Developmental delay	100.0 (14/14)
Exercise Intolerance	82.1 (23/28)	Chronic fatigue	92.9 (13/14)	Intellectual disability	85.7 (12/14)
Gastrointestinal problems	74.1 (20/27)	Sleep problems	85.7 (12/14)	Learning disability	85.7 (12/14)
Balance problems	67.9 (19/28)	Gastrointestinal problems	84.6 (11/13)	Chronic fatigue	78.6 (11/14)
Developmental Delay	65.4 (17/26)	Hyperlipidemia	75.0 (9/12)	Speech problems	71.4 (10/14)
Learning disability	65.4 (17/26)	Decreased vision	69.2 (9/13)	Balance Problems	71.4 (10/14)
Decreased vision	65.4 (17/26)	Peripheral neuropathy	69.2 (9/13)	Exercise intolerance	64.3 (9/14)
Speech problem	63.0 (17/27)	Dysautonomia	66.7 (8/12)	Gastrointestinal problems	64.3 (9/14)
Intellectual disability	61.5 (16/26)	Tinnitus	66.7 (8/12)	Decreased vision	61.5 (8/13)
Sleep problems	60.7 (17/28)	Balance problems	64.3 (9/14)	Epilepsy	50.0 (7/14)
Peripheral neuropathy	59.3 (16/27)	Headache	61.5 (8/13)	Optic nerve problems	50.0 (7/14)
Eye muscle problems	55.6 (15/27)	Diabetes	61.5 (8/13)	Eye muscle problems	50.0 (7/14)
Dysautonomia	53.8 (14/26)	Eye muscle problems	61.5 (8/13)	Peripheral Neuropathy	50.0 (7/14)
Hyperlipidemia	50.0 (13/26)	Dehydration	58.3 (7/12)	Dysautonomia	42.9 (6/14)
Optic nerve problems	48.1 (13/27)	Heart rhythm problems	58.3 (7/12)	Difficulty gaining weight	42.9 (6/14)
Headache	48.1 (13/27)	Hearing loss	57.1 (8/14)	Stroke	35.7 (5/14)
Epilepsy	46.2 (12/26)	Liver disease	54.5 (6/11)	Behavioral problems	35.7 (5/14)
Dehydration	46.2 (12/26)	Speech problems	53.8 (7/13)	Headache	35.7 (5/14)
Difficulty gaining weight	44.4 (12/27)	Mood disorder	53.8 (7/13)	Kidney disease	35.7 (5/14)
Hearing loss	42.9 (12/28)	Ptosis	53.8 (7/13)	Retinal problems	35.7 (5/14)
Kidney disease	42.3 (11/26)	Kidney disease	50.0 (6/12)	Sleep problems	35.7 (5/14)
Heart rhythm problems	42.3 (11/26)	Heart muscle problems	50.0 (6/12)	Dehydration	35.7 (5/14)
Ptosis	40.7 (11/27)	Optic nerve problems	46.2 (6/13)	Mood disorder	28.6 (4/14)

Mood disorder	40.7 (11/27)	Difficulty gaining weight	46.2 (6/13)	Autism-spectrum behaviors	28.6 (4/14)
Stroke	38.5 (10/26)	Epilepsy	41.7 (5/12)	Hyperlipidemia	28.6 (4/14)
Tinnitus	38.5 (10/26)	Stroke	41.7 (5/12)	Hearing loss	28.6 (4/14)
Retinal problems	38.5 (10/26)	Learning disability	41.7 (5/12)	Ptosis	28.6 (4/14)
Diabetes	37.0 (10/27)	Retinal problems	41.7 (5/12)	Heart rhythm problems	28.6 (4/14)
Heart muscle problems	34.6 (9/26)	Difficulty losing weight	41.7 (5/12)	Sleep apnea	21.4 (3/14)
Behavior problem	33.3 (9/27)	Intellectual disability	33.3 (4/12)	Heart muscle problems	21.4 (3/14)
Liver disease	32.0 (8/25)	Sleep apnea	33.3 (4/12)	Difficulty losing weight	21.4 (3/14)
Difficulty losing weight	30.8 (8/26)	Behavioral problems	30.8 (4/13)	Liver disease	14.3 (2/14)
Autism spectrum behavior	26.9 (7/26)	Developmental delay	25.0 (3/12)	Diabetes	14.3 (2/14)
Sleep apnea	26.9 (7/26)	Autism spectrum behavior	25.0 (3/12)	Tinnitus	14.3 (2/14)

[1] Respondents coded "likely to participate" responded "Would Participate" or "Likely to participate" in a trial aiming to treat the listed symptom.

[2] Nonrespondents on individual symptoms (maximum of 4 [26.7]% for Adults and 2 for Children [13.3]%] are excluded. Lower denominator (N) indicates the total number of respondents.

S1C Table. All patients, Adults and Children likely to participate[1] in a clinical trial if experienced symptom is targeted.

All (N=30)[2]		Adults (N=15)[2]		Children (N=15)[2]	
**Experienced symptom targeted by the trial**	**% (n)**	**Experienced symptom targeted by the trial**	**% (n)**	**Experienced symptom targeted by the trial**	**% (n)**
Muscle Weakness	100.0 (27/27)	Muscle Weakness	100.0 (14/14)	Muscle Weakness	100.0 (13/13)
Peripheral Neuropathy	100.0 (13/13)	Exercise Intolerance	100.0 (12/12)	Developmental Delay	100.0 (14/14)
Tinnitus	100.0 (7/7)	Peripheral Neuropathy	100.0 (9/9)	Learning Disability	100.0 (10/10)
Diabetes	100.0 (4/4)	Tinnitus	100.0 (7/7)	Dysautonomia	100.0 (4/4)
Stroke	100.0 (3/3)	Difficulty Gaining Weight	100.0 (5/5)	Optic Nerve Problems	100 (4/4)
Retinal Problems	100.0 (2/2)	Diabetes	100.0 (4/4)	Peripheral Neuropathy	100 (4/4)
Liver Disease	100.0 (1/1)	Stroke	100.0 (2/2)	Stroke	100.0 (1/1)
Delayed Milestones	93.8 (15/16)	Kidney Disease	100.0 (2/2)	Retinal Problems	100.0 (1/1)
Gastrointestinal Problems	90.9 (20/22)	Liver Disease	100.0 (1/1)	Gastrointestinal Problems	90 (9/10)
Dysautonomia	88.9 (8/9)	Retinal Problems	100.0 (1/1)	Intellectual Disability	90.0 (9/10)
Optic Nerve Problems	87.5 (7/8)	Chronic Fatigue	92.9 (13/14)	Behavioral Problems	80.0 (4/5)
Learning Disabilities	85.7 (12/14)	Gastrointestinal Problems	91.7 (11/12)	Eye Muscle Problems	80.0 (4/5)
Chronic Fatigue	85.2 (23/27)	Sleep Difficulties	90.9 (10/11)	Decreased Vision	77.8 (7/9)
Exercise Intolerance	83.3 (20/24)	Hyperlipidemia	87.5 (7/8)	Chronic Fatigue	76.9 (10/13)
Sleep Difficulties	83.3 (15/18)	Mood Disorder	85.7 (6/7)	Speech Problems	76.9 (10/13)
Kidney Disease	83.3 (5/6)	Eye Muscle Problems	83.3 (5/6)	Balance Problems	76.9 (10/13)
Eye Muscle Problems	81.8 (9/11)	Decreased Vision	81.8 (9/11)	Kidney Disease	75.0 (3/4)
Decreased Vision	80.0 (16/20)	Dysautonomia	80.0 (4/5)	Sleep Difficulties	71.4 (5/7)
Mood Disorder	80.0 (8/10)	Balance Problems	75.0 (9/12)	Exercise Intolerance	66.7 (8/12)
Intellectual Disability	78.6 (11/14)	Optic Nerve Problems	75.0 (3/4)	Epilepsy	66.7 (6/9)
Speech Problem	76.2 (16/21)	Speech Problems	75.0 (6/8)	Mood Disorder	66.7 (2/3)
Balance Problems	76.0 (19/25)	Heart Rhythm Problems	71.4 (5/7)	Ptosis	66.7 (2/3)

Hyperlipidemia	75.0 (9/12)	Epilepsy	66.7 (2/3)	Difficulty Gaining Weight	60.0 (6/10)
Difficulty Gaining weight	73.3 (11/15)	Headache	63.6 (7/11)	Dehydration	57.1 (4/7)
Behavioral Problems	70.0 (7/10)	Hearing Loss	62.5 (5/8)	Autism Spectrum Disorders	50.0 (2/4)
Epilepsy	66.7 (8/12)	Behavioral Problems	60.0 (3/5)	Hyperlipidemia	50.0 (2/4)
Heart Rhythm Problems	61.5 (8/13)	Dehydration	60.0 (6/10)	Hearing Loss	50.0 (1/2)
Hearing Loss	60.0 (6/10)	Ptosis	55.6 (5/9)	Sleep Apnea	50.0 (2/4)
Dehydration	58.8 (10/17)	Learning Disability	50.0 (2/4)	Heart Rhythm Problems	50.0 (3/6)
Ptosis	58.3 (7/12)	Difficulty Losing Weight	50.0 (2/4)	Headache	42.9 (3/7)
Headache	55.6 (10/18)	Heart Muscle Problems	50.0 (2/4)	Heart Muscle Problems	0.0 (0/2)
Sleep Apnea	50.0 (4/8)	Intellectual Disability	50.0 (2/4)	Difficulty Losing Weight	0.0 (0/1)
Autism Spectrum Behaviors	50.0 (2/4)	Sleep Apnea	50.0 (2/4)	Tinnitus	
Difficulty Losing Weight	40.0 (2/5)	Developmental Delay	50.0 (1/2)	Liver Disease	
Heart Muscle Problems	33.3 (2/6)	Autism Spectrum Disorder		Diabetes	

[1] Respondents coded "likely to participate" responded "Would Participate" or "Likely to participate" in a trial aiming to treat the listed symptom.

[2] Nonrespondents on individual symptoms and likely to participate (maximum 29 [58.0]% for all Adults and Children are excluded. Lower denominator (N) indicates the total number of respondents.

S1D Table. Symptoms most frequently selected by individuals in the top 3[1] that would prompt their participation in a clinical trial, among All patients, Adults and Children.

All (N=30)[2]		Adults (N=15)[2]		Children (N=15)[2]	
**Symptom**	**% (n)**	**Symptom**	**% (n)**	**Symptom**	**% (n)**
Muscle weakness	53.6 (15/28)	Muscle weakness	50.0 (7/14)	Developmental delay	78.6 (11/14)
Developmental delay	39.3 (11/28)	Exercise Intolerance	42.9 (6/14)	Muscle weakness	57.1 (8/14)
Chronic fatigue	32.1 (9/28)	Chronic fatigue	42.9 (6/14)	Epilepsy	35.7 (5/14)
Epilepsy	25.0 (7/28)	Gastrointestinal	28.6 (4/14)	Chronic fatigue	21.4 (3/14)
Exercise Intolerance	21.4 (6/28)	Epilepsy	14.3 (2/14)	Speech problem	21.4 (3/14)
Gastrointestinal	21.4 (6/28)	Headache	14.3 (2/14)	Intellectual disability	21.4 (3/14)
Intellectual Disability	14.3 (4/28)	Balance problems	14.3 (2/14)	Kidney disease	14.3 (2/14)
Peripheral neuropathy	14.3 (4/28)	Peripheral neuropathy	14.3 (2/14)	Peripheral neuropathy	14.3 (2/14)
Speech problems	10.7 (3/28)	Difficulty Sleeping	14.3 (2/14)	Gastrointestinal	14.3 (2/14)
Headache	7.1 (2/28)	Stroke	7.1 (1/14)	Learning disability	7.1 (1/14)
Kidney disease	7.1 (2/28)	Intellectual Disability	7.1 (1/14)	Optic nerve problems	7.1 (1/14)
Eye muscle problems	7.1 (2/28)	Diabetes	7.1 (1/14)	Eye muscle problems	7.1 (1/14)
Balance problems	7.1 (2/28)	Dysautonomia	7.1 (1/14)	Exercise intolerance	0 (0/14)
Difficulty Sleeping	7.1 (2/28)	Hearing Loss	7.1 (1/14)	Stroke	0 (0/14)
Stroke	3.6 (1/28)	Eye Muscle Problems	7.1 (1/14)	Behavior problem	0 (0/14)
Learning disability	3.6 (1/28)	Heart muscle problems	7.1 (1/14)	Mood disorder	0 (0/14)
Diabetes	3.6 (1/28)	Heart rhythm problems	7.1 (1/14)	Headache	0 (0/14)
Dysautonomia	3.6 (1/28)	Difficulty Losing Weight	7.1 (1/14)	Autism spectrum	0 (0/14)
Hearing Loss	3.6 (1/28)	Developmental Delay	0 (0/14)	Dehydration	0 (0/14)
Optic Nerve problems	3.6 (1/28)	Speech Problems	0 (0/14)	Liver disease	0 (0/14)
Heart muscle problems	3.6 (1/28)	Behavioral problems	0 (0/14)	Diabetes	0 (0/14)
Heart rhythm problems	3.6 (1/28)	Mood Disorder	0 (0/14)	Cholesterol	0 (0/14)
Difficulty losing weight	3.6 (1/28)	Learning Disability	0 (0/14)	Dysautonomia	0 (0/14)

Behavior problem	0 (0/28)	Autism Spectrum	0 (0/14)	Hearing loss	0 (0/14)
Mood disorder	0 (0/28)	Dehydration	0 (0/14)	Tinnitus	0 (0/14)
Autism spectrum	0 (0/28)	Liver disease	0 (0/14)	Decrease vision	0 (0/14)
Dehydration	0 (0/28)	Kidney Disease	0 (0/14)	Retinal problem	0 (0/14)
Liver disease	0 (0/28)	Cholesterol	0 (0/14)	Ptosis	0 (0/14)
Cholesterol	0 (0/28)	Tinnitus	0 (0/14)	Balance problem	0 (0/14)
Tinnitus	0 (0/28)	Decreased Vision	0 (0/14)	Difficulty sleeping	0 (0/14)
Decreased vision	0 (0/28)	Retinal Problems	0 (0/14)	Sleep apnea	0 (0/14)
Retinal problem	0 (0/28)	Optic Nerve Problems	0 (0/14)	Heart muscle problems	0 (0/14)
Ptosis	0 (0/28)	Ptosis	0 (0/14)	Heart rhythm problems	0 (0/14)
Sleep apnea	0 (0/28)	Sleep Apnea	0 (0/14)	Difficulty gaining weight	0 (0/14)
Difficulty gaining weight	0 (0/28)	Difficulty gaining weight	0 (0/14)	Difficulty losing weight	0 (0/14)

[1] Respondents selected 3 symptoms from 35 symptoms listed
[2] Nonrespondents on individual symptoms (maximum 2 [6.7%] for all patients are excluded. Lower denominator (N) indicates the total number of respondents
