# Supplementary material for: Mitochondrial disease patient motivations and barriers to participate in clinical trials
Source: PLoS One. 2018 May 17;13(5):e0197513. doi: 10.1371/journal.pone.0197513 (PMC5957366; doi:10.1371/journal.pone.0197513)
Supplement: S2 Table — A. Symptoms Experienced by All patients, Adults and Children. 1Nonrespondents on individual symptoms (maximum for 16 [5.5%] Adults and 15 [5.2%] for Children) are excluded. Lower denominator (N) indicates the total number of respondents. B. All patients, Adults and Children likely to participate1 in a clinical trial, by symptom targeted. 1Respondents coded “likely to participate” responded “Would Participate” or “Likely to participate” in a trial aiming to treat the listed symptom. 2Nonrespondents on individual symptoms (maximum 27 [16.0%] for Adults and 14 [4.8%] for Children) are excluded. Lower denominator (N) indicates the total number of respondents. C. All patients, Adults and Children likely to participate1 in a clinical trial if experienced symptom is targeted. 1Respondents coded “likely to participate” responded “Would Participate” or “Likely to participate” in a trial aiming to treat the listed symptom. 2Nonrespondents on individual symptoms (maximum 258 [88.9%] for Adults and Children) are excluded. Lower denominator (N) indicates the total number of respondents. D. Symptoms most frequently selected by individuals in the top 31 that would prompt their participation in a clinical trial, among All patients, Adults and Children. 1Participants were asked to select, from a list of 35 symptoms, the top 3 that would prompt their participation in a clinical trial. 2Nonrespondents on individual symptoms (maximum 0 [0.0%] for Adults and 0 [0.0%] for Children) are excluded. Lower denominator (N) indicates the total number of respondents. (PDF) [file pone.0197513.s003.pdf]

S2A Table. Symptoms Experienced by All patients, Adults and Children.

All (N=290)[1]		Adults (N=169)[1]		Children (N=121)[1]	
**Symptom**	**% (n)**	**Symptom**	**% (n)**	**Symptom**	**% (n)**
Muscle weakness	95.4 (270/283)	Chronic fatigue	94.6 (156/165)	Muscle weakness	98.3 (116/118)
Chronic fatigue	95.1 (269/283)	Muscle weakness	93.3 (154/165)	Exercise intolerance	97.5 (115/118)
Exercise intolerance	94.7 (268/283)	Exercise intolerance	92.7 (153/165)	Chronic fatigue	95.8 (113/118)
GI Problems	79.6 (223/280)	Balance problems	79.6 (129/162)	Gastrointestinal problems	87.2 (102/117)
Balance Problems	78.8 (219/278)	Gastrointestinal problems	74.2 (121/163)	Delayed milestones	86.3 (101/117)
Sleep Problems	68.7 (191/278)	Sleep problems	72.0 (116/161)	Speech problems	77.8 (91/117)
Decreased Vision	64.7 (180/278)	Decreased vision	72.0 (116/161)	Balance problems	77.6 (90/116)
Headache	63.3 (176/278)	Headache	65.4 (106/162)	Learning disability	73.9 (85/115)
Dehydration	56.7 (157/277)	Peripheral neuropathy	58.9 (93/158)	Dehydration	68.7 (79/115)
Peripheral Neuropathy	55.9 (151/270)	Eye muscle problems	57.9 (92/159)	Intellectual disability	65.5 (76/115)
Eye Muscle Problems	55.3 (151/273)	Ptosis	56.6 (90/159)	Sleep problems	64.1 (75/117)
Delayed milestones	55.2 (149/270)	Mood disorder	50.9 (83/163)	Headache	60.3 (70/116)
Speech Problems	55.0 (155/282)	Tinnitus	50.0 (80/160)	Behavioral problem	57.9 (66/114)
Mood Disorder	51.1 (142/278)	Difficulty losing weight	49.4 (77/156)	Dysautonomia	57.5 (65/113)
Ptosis	50.9 (139/273)	Dehydration	48.2 (78/162)	Decreased vision	54.7 (64/117)
Dysautonomia	48.1 (124/258)	Hyperlipidemia	44.8 (73/163)	Difficulty gaining weight	54.4 (62/114)
Learning Disability	43.5 (120/276)	Heart rhythm problems	44.2 (72/163)	Peripheral neuropathy	51.8 (58/112)
Difficulty Gaining weight	40.6 (112/276)	Hearing loss	41.7 (68/163)	Eye muscle problems	51.8 (59/114)
Hearth Rhythm Problems	39.2 (109/278)	Dysautonomia	40.7 (59/145)	Mood disorder	51.3 (59/115)
Difficulty losing weight	38.9 (102/262)	Speech problems	38.8 (64/165)	Epilepsy	45.8 (54/118)
Intellectual Disability	38.7 (108/279)	Delayed milestones	31.4 (48/153)	Ptosis	43.0 (49/114)
Sleep Apnea	35.4 (97/274)	Difficulty gaining weight	30.9 (50/162)	Sleep apnea	42.1 (48/114)
Tinnitus	34.3 (93/271)	Sleep apnea	30.6 (49/160)	Autism spectrum behavior	39.1 (45/115)

Behavioral Problems	34.3 (95/277)	Optic nerve problems	28.5 (45/158)	Heart rhythm problems	32.2 (37/115)
Hearing Loss	33.5 (93/278)	Retinal problems	26.0 (41/158)	Optic nerve problems	23.7 (27/114)
Hyperlipidemia	33.3 (92/276)	Heart muscle problems	22.0 (35/159)	Difficulty losing weight	23.6 (25/106)
Epilepsy	31.2 (88/282)	Learning disability	21.7 (35/161)	Heart muscle problems	22.8 (26/114)
Optic Nerve Problems	26.5 (72/272)	Diabetes	21.6 (35/162)	Hearing loss	21.7 (25/115)
Heart Muscle Problems	22.3 (61/273)	Epilepsy	20.7 (34/164)	Hyperlipidemia	16.8 (19/113)
Autism Spectrum Disorder	22.0 (60/273)	Intellectual disability	19.6 (32/163)	Retinal problems	15.8 (18/114)
Retinal Problems	21.7 (59/272)	Behavioral problem	17.8 (29/163)	Stroke	13.0 (15)
Diabetes	14.7 (41/278)	Stroke	12.4 (20/161)	Liver disease	12.8 (15/117)
Stroke	12.7 (35/276)	Liver disease	11.7 (19/162)	Tinnitus	11.7 (13/111)
Liver Disease	12.2 (34/279)	Kidney disease	10.6 (17/161)	Kidney disease	6.0 (7/116)
Kidney Disease	8.7 (24/277)	Autism spectrum behavior	9.5 (15/158)	Diabetes	5.2 (6/116)

[1]Nonrespondents on individual symptoms (maximum for 16 [5.5%] Adults and 15 [5.2%] for Children) are excluded. Lower denominator (N) indicates the total number of respondents.

S2B Table. All patients, Adults and Children likely to participate[1] in a clinical trial, by symptom targeted.

All (N=290)[2]		Adults (N=169)[2]		Children (N=121)[2]	
**Symptom targeted by the trial**	**% (n)**	**Symptom targeted by the trial**	**% (n)**	**Symptom targeted by the trial**	**% (n)**
Muscle weakness	91.0 (244/268)	Chronic fatigue	94.2 (146/155)	Muscle weakness	88.6 (101/114)
Chronic fatigue	87.7 (236/269)	Exercise intolerance	94.2 (146/155)	Chronic fatigue	79.0 (90/114)
Exercise intolerance	87.3 (234/268)	Muscle weakness	92.9 (143/154)	Exercise intolerance	77.9 (88/113)
Gastrointestinal problems	73.0 (192/263)	Balance problems	74.0 (111/150)	Gastrointestinal problems	75.9 (85/112)
Balance problems	72.9 (188/258)	Eye muscle problems	71.4 (105/147)	Delayed milestones	73.5 (83/113)
Peripheral neuropathy	65.4 (166/254)	Peripheral neuropathy	71.0 (103/145)	Balance problems	71.3 (77/108)
Sleep problems	64.8 (169/261)	Gastrointestinal problems	70.9 (107/151)	Learning disability	71.2 (79/111)
Eye muscle problems	62.6 (161/257)	Decreased vision	67.8 (101/149)	Intellectual disability	68.8 (75/109)
Decreased vision	60.7 (159/262)	Sleep problems	66.9 (101/151)	Speech problems	68.5 (76/111)
Headache	57.1 (149/261)	Ptosis	63.8 (95/149)	Sleep problems	61.8 (68/110)
Ptosis	56.2 (146/260)	Headache	58.0 (87/150)	Dehydration	60.7 (68/112)
Dehydration	56.1 (148/264)	Difficulty losing weight	56.1 (83/148)	Dysautonomia	58.9 (63/107)
Speech problems	53.7 (139/259)	Heart rhythm problems	54.1 (79/146)	Peripheral neuropathy	57.8 (63/109)
Dysautonomia	52.0 (131/252)	Hyperlipidemia	53.0 (79/149)	Headache	55.9 (62/111)
Delayed milestones	50.8 (131/258)	Dehydration	52.6 (80/152)	Behavioral problem	52.3 (57/109)
Learning disability	50.4 (130/258)	Hearing loss	52.4 (76/145)	Mood disorder	51.8 (57/110)
Heart rhythm problems	50.0 (128/256)	Optic nerve problems	51.4 (76/148)	Decreased vision	51.3 (58/113)
Sleep apnea	49.6 (129/260)	Retinal problems	51.0 (73/143)	Eye muscle problems	50.9 (56/110)
Intellectual disability	49.2 (126/256)	Heart muscle problems	50.7 (75/148)	Sleep apnea	50.9 (57/112)
Mood disorder	49.0 (127/259)	Sleep apnea	48.7 (72/148)	Epilepsy	48.7 (54/111)
Optic nerve problems	48.5 (126/260)	Tinnitus	48.6 (71/146)	Difficulty gaining weight	48.7 (54/111)
Heart muscle problems	46.9 (121/258)	Mood disorder	47.0 (70/149)	Autism spectrum behavior	46.4 (51/110)
Difficulty losing weight	46.5 (120/258)	Dysautonomia	46.9 (68/145)	Ptosis	46.0 (51/111)
Hearing loss	46.1 (118/256)	Diabetes	45.1 (64/142)	Optic nerve problems	44.6 (50/112)

Retinal problems	45.5 (116/255)	Speech problems	42.6 (63/148)	Heart rhythm problems	44.6 (49/110)
Hyperlipidemia	45.4 (118/260)	Kidney disease	40.5 (60/148)	Heart muscle problems	41.8 (46/110)
Difficulty gaining weight	41.0 (105/256)	Stroke	38.1 (56/147)	Retinal problems	38.4 (43/112)
Behavioral problem	40.9 (105/257)	Liver disease	37.2 (55/148)	Hearing loss	37.8 (42/111)
Tinnitus	40.5 (104/257)	Difficulty gaining weight	35.2 (51/145)	Hyperlipidemia	35.1 (39/111)
Diabetes	40.2 (101/251)	Intellectual disability	34.7 (51/147)	Stroke	34.8 (39/112)
Epilepsy	40.2 (104/259)	Learning disability	34.7 (51/147)	Diabetes	33.9 (37/109)
Stroke	36.7 (95/259)	Epilepsy	33.8 (50/148)	Difficulty losing weight	33.6 (37/110)
Kidney disease	36.2 (93/257)	Delayed milestones	33.1 (48/145)	Liver disease	31.8 (35/110)
Autism spectrum behavior	34.9 (89/255)	Behavioral problem	32.4 (48/148)	Kidney disease	30.3 (33/109)
Liver disease	34.9 (90/258)	Autism spectrum behavior	26.2 (38/145)	Tinnitus	29.7 (33/111)

[1] Respondents coded “likely to participate” responded “Would Participate” or “Likely to participate” in a trial aiming to treat the listed symptom.

[2] Nonrespondents on individual symptoms (maximum 27 [16.0%] for Adults and 14 [4.8%] for Children) are excluded. Lower denominator (N) indicates the total number of respondents

S2C Table. All patients, Adults and Children likely to participate[1] in a clinical trial if experienced symptom is targeted.

All (N=290)[2]		Adults (N=169)[2]		Children (N=121)[2]	
**Experienced symptom targeted by the trial**	**% (n)**	**Experienced symptom targeted by the trial**	**% (n)**	**Experienced symptom targeted by the trial**	**% (n)**
Muscle Weakness	93.0 (239/257)	Kidney Disease	100.0 (16/16)	Diabetes	100.0 (4/4)
Chronic Fatigue	90.3 (232/257)	Exercise Intolerance	97.2 (140/144)	Muscle Weakness	89.3 (100/112)
Exercise Intolerance	89.4 (227/254)	Chronic Fatigue	96.6 (143/148)	Intellectual Disability	87.5 (63/72)
Diabetes	86.5 (32/37)	Muscle Weakness	95.9 (139/145)	Learning Disability	86.6 (71/82)
Kidney Disease	86.4 (19/22)	Ptosis	89.4 (76/85)	Stroke	85.7 (12/14)
Intellectual Disability	84.3 (86/102)	Eye Muscle Problems	88.6 (78/88)	Optic Nerve Problems	84.6 (22/26)
Optic Nerve Problems	84.3 (57/68)	Peripheral Neuropathy	88.4 (76/86)	Dysautonomia	83.6 (51/61)
Dysautonomia	83.8 (97/116)	Gastrointestinal Problems	85.8 (97/113)	Gastrointestinal Problems	82.5 (80/97)
Peripheral Neuropathy	83.6 (116/139)	Sleep Apnea	85.4 (41/48)	Chronic Fatigue	81.7 (89/109)
Balance Problems	83.5 (168/201)	Diabetes	84.8 (28/33)	Delayed Milestones	81.6 (80/98)
Ptosis	82.0 (107/131)	Difficulty Losing Weight	84.7 (61/72)	Balance Problems	81.2 (69/85)
Eye Muscle Problems	81.7 (118/144)	Dysautonomia	83.6 (46/55)	Autism Spectrum Behaviors	81.0 (34/42)
Learning Disability	81.4 (94/116)	Optic Nerve Problems	83.3 (35/42)	Speech Problem	80.5 (70/87)
Hearing Loss	81.0 (68/84)	Hearing Loss	82.5 (52/63)	Exercise Intolerance	79.1 (87/110)
Sleep Apnea	81.0 (74/91)	Balance Problems	82.5 (99/120)	Sleep Difficulties	78.6 (55/70)
Stroke	79.6 (27/34)	Decreased Vision	82.1 (87/106)	Hearing Loss	76.2 (16/21)
Difficulty Losing Weight	79.4 (76/96)	Heart Rhythm Problems	80.6 (54/65)	Heart Rhythm Problems	75.8 (25/33)
Sleep Difficulties	79.2 (140/177)	Sleep Difficulties	79.4 (85/107)	Peripheral Neuropathy	75.5 (40/53)
Heart Rhythm Problems	79.1 (79/100)	Retinal Problems	78.4 (29/37)	Epilepsy	74.0 (37/50)
Gastrointestinal Problems	79.0 (117/148)	Intellectual Disability	76.7 (23/30)	Sleep Apnea	73.3 (33/45)
Decreased Vision	76.5 (127/166)	Heart Muscle Problems	75.8 (25/33)	Headache	72.7 (48/66)

Autism Spectrum Behaviors	76.4 (42/55)	Dehydration	75.7 (56/74)	Retinal Problems	72.2 (13/18)
Retinal Problems	76.4 (42/55)	Stroke	75.0 (15/20)	Difficulty Gaining Weight	72.1 (44/61)
Speech Problem	76.4 (113/148)	Headache	74.7 (74/99)	Dehydration	71.4 (55/77)
Delayed Milestones	76.1 (108/142)	Hyperlipidemia	74.3 (52/70)	Heart Muscle Problems	70.8 (17/24)
Headache	73.9 (122/165)	Tinnitus	71.6 (53/74)	Eye Muscle Problems	70.2 (40/57)
Heart Muscle Problems	73.7 (42/57)	Speech Problem	70.5 (43/61)	Liver Disease	69.2 (9/13)
Dehydration	73.5 (111/151)	Behavioral Problems	70.4 (19/27)	Behavioral Problems	68.8 (44/64)
Hyperlipidemia	72.4 (63/87)	Epilepsy	69.7 (23/33)	Ptosis	67.4 (31/46)
Epilepsy	72.3 (60/83)	Learning Disability	67.6 (23/34)	Decreased Vision	66.7 (40/60)
Tinnitus	69.8 (60/86)	Mood Disorder	67.5 (54/80)	Mood Disorder	64.9 (37/57)
Behavioral Problems	69.2 (63/91)	Delayed Milestones	63.6 (28/44)	Hyperlipidemi a	64.7 (11/17)
Difficulty Gaining Weight	66.7 (72/108)	Liver Disease	63.2 (12/19)	Difficulty Losing Weight	62.5 (15/24)
Mood Disorder	66.4 (91/137)	Autism Spectrum Behaviors	61.5 (8/13)	Tinnitus	58.3 (7/12)
Liver Disease	65.6 (21/32)	Difficulty Gaining Weight	59.6 (28/47)	Kidney Disease	50.0 (3/6)

Respondents coded “likely to participate” responded “Would Participate” or “Likely to participate” in a trial aiming to treat the listed symptom.

[2] **Nonrespondents on individual symptoms (maximum 258 [88.9%] for Adults and Children) are excluded. Lower denominator (N) indicates the total number of respondents.**

S2D Table. Symptoms most frequently selected by individuals in the top 3[1] that would prompt their participation in a clinical trial, among All patients, Adults and Children.

All (N=290)[2]		Adults (N=169)[2]		Children (N=121)[2]	
**Symptom**	**% (n)**	**Symptom**	**% (n)**	**Symptom**	**% (n)**
Muscle weakness	43.1 (125/290)	Chronic fatigue	52.7 (89/169)	Muscle weakness	40.5 (49/121)
Chronic fatigue	41.4 (120/290)	Muscle weakness	45.0 (76/169)	Chronic fatigue	25.6 (31/121)
Exercise intolerance	23.8 (69/290)	Exercise intolerance	32.5 (55/169)	Gastrointestinal problems	24.0 (29/121)
Dysautonomia	17.9 (62/290)	Eye muscle problems	17.8 (30/169)	Dysautonomia	23.1 (28/121)
Gastrointestinal problems	17.9 (52/290)	Dysautonomia	14.2 (24/169)	Epilepsy	19.0 (23/121)
Epilepsy	10.7 (31/290)	Ptosis	14.2 (24/169)	Speech problems	12.4 (15/121)
Eye muscle problems	10.7 (31/290)	Gastrointestinal problems	13.6 (23/169)	Exercise intolerance	11.6 (14/121)
Ptosis	8.6 (25/290)	Peripheral neuropathy	10.7 (18/169)	Intellectual disability	10.7 (13/121)
Peripheral neuropathy	7.2 (21/290)	Decreased vision	8.3 (14/169)	Autism spectrum behavior	9.9 (12/121)
Imbalance	6.9 (20/290)	Headache	8.3 (14/169)	Learning disability	9.9 (12/121)
Decreased vision	6.2 (18/290)	Imbalance	7.7 (13/169)	Behavioral problem	9.1 (11/121)
Speech problem	6.2 (18/290)	Difficulty losing weight	4.7 (8/169)	Delayed milestones	8.3 (10/121)
Headache	5.5 (16/290)	Epilepsy	4.7 (8/169)	Difficulty gaining weight	8.3 (10/121)
Sleep problems	5.2 (15/290)	Sleep problems	4.1 (7/169)	Sleep problems	6.6 (8/121)
Intellectual disability	5.2 (15/290)	Retinal problems	4.1 (7/169)	Balance problems	5.8 (7/121)
Difficulty gaining weight	4 .5(13/290)	Heart rhythm problems	3.6 (6/169)	Heart rhythm problems	4.1 (5/121)
Autism spectrum behavior	4.1 (12/290)	Optic nerve problems	3.6 (6/169)	Liver disease	4.1 (5/121)
Learning disability	4.1 (12/290)	Dehydration	3.0 (5/169)	Mood disorder	4.1 (5/121)
Retinal problems	4.1 (12/290)	Diabetes	3.0 (5/169)	Retinal problems	4.1 (5/121)
Behavioral problem	3.8 (11/290)	Heart muscle problems	3.0 (5/169)	Decreased vision	3.3 (4/121)
Heart rhythm problems	3.4 (11/290)	Hearing loss	2.4 (4/169)	Hearing loss	3.3 (4/121)
Delayed milestones	3.4 (10/290)	Stroke	2.4 (4/169)	Heart muscle problems	3.3 (4/121)
Difficulty losing weight	3.4 (10/290)	Difficulty gaining weight	1.8 (3/169)	Optic nerve problems	3.3 (4/121)
Optic nerve problems	3.4 (10/290)	Speech problems	1.8 (3/169)	Sleep apnea	3.3 (4/121)

Heart muscle problems	3.1 (9/290)	Tinnitus	1.8 (3/169)	Peripheral neuropathy	2.5 (3/121)
Hearing loss	2.8 (8/290)	Intellectual disability	1.2 (2/169)	Dehydration	1.7 (2/121)
Dehydration	2.4 (7/290)	Mood disorder	1.2 (2/169)	Difficulty losing weight	1.7 (2/121)
Mood disorder	2.4 (7/290)	Hyperlipidemia	0.6 (1/169)	Headache	1.7 (2/121)
Diabetes	2.1 (6/290)	Liver disease	0.6 (1/169)	Stroke	1.7 (2/121)
Liver disease	2.1 (6/290)	Sleep apnea	0.6 (1/169)	Diabetes	0.8 (1/121)
Stroke	2.1 (6/290)	Autism spectrum behavior	0.0 (0/169)	Eye muscle problems	0.8 (1/121)
Sleep apnea	1.7 (5/290)	Behavioral problem	0.0 (0/169)	Hyperlipidemia	0.8 (1/121)
Tinnitus	1.0 (3/290)	Delayed milestones	0.0 (0/169)	Kidney disease	0.8 (1/121)
Hyperlipidemia	0.7 (2/290)	Kidney disease	0.0 (0/169)	Ptosis	0.8 (1/121)
Kidney disease	0.3 (1/290)	Learning disability	0.0 (0/169)	Tinnitus	0. (0.0/121)

[1]Participants were asked to select, from a list of 35 symptoms, the top 3 that would prompt their participation in a clinical trial.

[2] Nonrespondents on individual symptoms (maximum 0 [0.0%] for Adults and 0 [0.0%] for Children) are excluded. Lower denominator (N) indicates the total number of respondents.
