# Supplementary material for: Mitochondrial disease patient motivations and barriers to participate in clinical trials
Source: PLoS One. 2018 May 17;13(5):e0197513. doi: 10.1371/journal.pone.0197513 (PMC5957366; doi:10.1371/journal.pone.0197513)
Supplement: S3 Table — 1Nonrespondents on individual symptoms are excluded. (PDF) [file pone.0197513.s004.pdf]

S3 Table. Symptom Severity in Adults and Children.

Adults (N=169)[1]					Children (N=121)[1]				
Symptom (N analyzed)	% Very severe	% Severe	% Moderate	% Mild	Symptom (N analyzed)	% Very severe	% Severe	% Moderate	% Mild
Chronic fatigue (156)	10.9	39.4	35.2	9.1	Muscle weakness (116)	23.7	26.3	33.1	15.3
Muscle weakness (154)	9.1	33.3	33.9	17.0	Exercise intolerance (115)	27.1	27.1	33.1	10.2
Exercise intolerance (115)	27.3	37.6	21.2	6.7	Chronic fatigue (113)	14.4	21.2	39.0	21.2
Balance problems (129)	6.8	16.1	30.3	26.5	Gastrointestinal problems (102)	24.8	17.1	27.4	18.0
Gastrointestinal problems (121)	11.04	17.2	30.7	15.3	Delayed milestones (101)	29.1	20.5	23.1	13.7
Sleep problems (116)	14.3	13.7	24.8	19.3	Speech problems (91)	30.8	11.1	16.2	19.7
Decreased vision (116)	7.5	16.2	20.5	28.0	Balance problems (90)	26.7	13.8	18.1	19.0
Headache (106)	4.9	17.3	24.1	19.1	Learning disability (85)	22.6	11.3	26.1	13.9
Peripheral neuropathy (93)	5.7	14.6	20.3	18.4	Dehydration (79)	2.6	17.4	23.5	25.2
Eye muscle problems (92)	11.3	18.2	12.0	16.4	Intellectual disability (76)	18.1	12.9	19.8	14.7
Ptosis (90)	15.1	15.7	8.8	17.0	Sleep problems (75)	17.1	12.8	17.1	17.1
Mood disorder (83)	1.8	3.1	16.0	30.1	Headache (70)	6.9	9.5	21.6	22.4
Tinnitus (80)	6.3	8.1	11.9	23.8	Behavioral problem (66)	1.8	9.7	23.7	22.8
Difficulty losing weight (77)	10.3	10.3	17.3	11.5	Dysautonomia (65)	17.7	8.0	21.2	10.6
Dehydration (78)	3.7	8.6	16.1	19.8	Decreased vision (64)	12.0	7.7	18.8	16.2
Hyperlipidemia (73)	5.5	6.1	17.8	15.3	Difficulty gaining weight (62)	5.3	10.5	21.1	17.5
Heart rhythm problems (72)	1.8	4.3	12.9	25.2	Peripheral neuropathy (58)	11.6	8.0	15.2	17.0
Hearing loss (68)	4.3	6.8	12.3	18.4	Eye muscle problems (59)	8.8	7.9	20.2	14.9
Dysautonomia (59)	5.5	10.3	12.4	12.4	Mood disorder (59)	2.6	5.2	20.0	23.5
Speech problems (64)	0.6	1.8	10.3	26.1	Epilepsy or seizures (54)	11.0	10.2	8.5	16.1
Delayed milestones (48)	2.0	4.6	11.8	13.1	Ptosis (49)	4.4	7.9	13.2	17.5
Difficulty gaining weight (50)	6.2	5.6	8.0	11.1	Sleep apnea (48)	7.9	8.8	8.8	16.7
Sleep apnea (49)	3.8	6.3	12.5	8.13	Autism spectrum (45)	4.4	10.4	11.3	13.0
Optic nerve problems (45)	2.5	5.7	8.9	11.4	Heart rhythm problems (37)	2.6	2.6	13.0	13.9
Retinal problems (41)	1.9	3.2	9.5	11.4	Optic nerve problems (27)	8.8	5.3	5.3	4.4

Heart muscle problems (35)	0.6	3.1	5.7	12.6	Difficulty losing weight (25)	1.9	4.7	6.6	10.4
Learning disability (35)	1.2	1.2	9.3	9.9	Heart muscle problems (26)	1.8	0.9	4.4	15.8
Diabetes (35)	1.2	4.9	6.8	8.6	Hearing loss (25)	6.1	3.5	0	12.2
Epilepsy or seizures (34)	0.6	0.6	4.9	14.6	Hyperlipidemia (19)	0	3.5	3.5	9.7
Intellectual disability (32)	0.6	1.2	4.9	12.9	Retinal problems (18)	2.6	2.6	5.3	5.3
Behavioral problem (29)	0	1.8	5.5	10.4	Liver disease (15)	0.9	5.1	0.9	6.0
Stroke (20)	0.6	0.6	2.5	8.6	Stroke (15)	0.9	3.5	3.5	5.2
Liver disease (19)	0.6	0.6	2.5	8.0	Tinnitus (13)	0.9	0.9	1.8	8.1
Kidney disease (17)	0	1.2	3.1	6.2	Kidney disease (7)	0.9	1.7	1.7	1.7
Autism spectrum (15)	0	0.6	2.5	6.3	Diabetes (6)	1.7	0.9	0.9	1.7

[1] **Nonrespondents on individual symptoms are excluded**
