# Supplementary material for: Mitochondrial disease patient motivations and barriers to participate in clinical trials
Source: PLoS One. 2018 May 17;13(5):e0197513. doi: 10.1371/journal.pone.0197513 (PMC5957366; doi:10.1371/journal.pone.0197513)
Supplement: S4 Table — (PDF) [file pone.0197513.s005.pdf]

S4 Table. Likelihood to participate in clinical trials with differing design and other features in All patients (N=30), Adults (N=15) and Children (N=15).

Questions		% (n)		
**A. Drug Therapy**				
**i. How likely would you/your child be to participate in a clinical trial if it involves:**				
		All N=30	Adults N=15	Children N=15
Taking a new drug that has never been used before on people		20.0 (6/30)	20.0 (3/15)	20.0 (3/15)
Taking a drug that has been used for other purposes, but not for mitochondrial disease		70.0 (21/30)	66.7 (10/15)	73.3 (11/15)
Taking a vitamin		96.7 (29/30)	93.3 (14/15)	100.0 (15/15)
Taking an antioxidant		90.0 (27/30)	93.3 (14/15)	86.7 (13/15)
Taking a natural supplement that is available at health food stores (ie. GNC)		96.7 (29/30)	93.3 (14/15)	100.0 (15/15)
Taking a plant-derived product		83.3 (25/30)	93.3 (14/15)	73.3 (11/15)
Taking a food product		93.3 (28/30)	93.3 (14/15)	93.3 (14/15)
Participating in an exercise test		72.4 (21/29)	80.0 (12/15)	64.3 (9/14)
Making no changes to your current medication		66.7 (20/30)	73.3 (11/15)	60.0 (9/15)
Stopping one of your current medications		50.0 (15/30)	60.0 (9/15)	40.0 (6/15)
Stopping all of your current medications		20.0 (6/30)	13.3 (2/15)	26.7 (4/15)
Changing your diet		70.0 (21/30)	60.0 (9/15)	80.0 (12/15)
[1]Nonrespondents on individual questions (maximum 1 [3.3%] for All, 0 [0.0%] for Adults and 1 [6.7%] for Children) are excluded.				
**ii. How likely would you/your child be to participate in a clinical trial if the drug:**				
		All N=30	Adults N=15	Children N=15
Is a pill		83.3 (25/30)	86.7 (13/15)	80.0 (12/15)
Is an injection		46.7 (14/30)	40.0 (6/15)	53.3 (8/15)
Has to be taken one time a day		93.1 (27/29)	93.3 (14/15)	92.9 (13/14)
Has to be taken two times a day		90.0 (27/30)	86.7 (13/15)	93.3 (14/15)
Has to be taken three times a day		73.3 (22/30)	66.7 (10/15)	80.0 (12/15)
Has to be taken four or more times a day		63.3 (19/30)	53.3 (8/15)	73.3 (11/15)
Can be self administered		69.0 (20/29)	66.7 (10/15)	71.4 (10/14)
Has to be given by a nurse		43.3 (13/30)	26.7 (4/15)	60.0 (9/15)
Has to be given at the hospital		30.0 (9/30)	20.0 (3/15)	40.0 (6/15)
[1]Nonrespondents on individual questions (maximum 1 [3.3%] for all, 0 [0.0%] for Adults and 1 [6.7%] for Children) are excluded.				
**iii. If you/your child were enrolled in a clinical trial:**				
		All N=30	Adults N=15	Children N=15
And the same drug became widely available to all people would you…	Quit the clinical trial	6.7 (2/30)	6.7 (1/15)	6.7 (1/15)
	Stay in the clinical trial	93.3 (28/30)	93.3 (14/15)	93.3 (14/15)
And a comparable drug became widely	Quit the clinical trial	3.4 (1/29)	6.7 (1/15)	0.0 (0/14)

available to all people, would you…	Stay in the clinical trial	96.6 (28/29)	93.3 (14/15)	100.0 (14/14)
And a promising new but completely unrelated drug became widely available to all people would you…	Quit the clinical trial	17.2 (5/29)	26.7 (4/15)	7.1 (1/14)
	Stay in the clinical trial	82.8 (24/29)	73.3 (11/15)	92.9 (13/14)
And you had progression of your disease symptoms while enrolled, would you…	Quit the clinical trial	53.3 (16/30)	40.0 (6/15)	66.7 (10/15)
	Stay in the clinical trial	46.7 (14/30)	60.0 (9/15)	33.3 (5/15)

[1]Nonrespondents on individual questions (maximum 1 [3.3%] for All, 0 [0.0%] for Adults and 1 [6.7%] for Children) are excluded.

B. Goal of the Study

i. How likely would you/your child be to participate in a clinical trial if it is supposed to help with:

	All N=30	Adults N=15	Children N=15
Only one of the symptoms that you/your child experiences from the mitochondrial disease	79.3 (23/29)	66.7 (10/15)	92.9 (13/14)
Multiple symptoms that you/your child experience but not all of the symptoms	93.1 (27/29)	86.7 (13/15)	100.0 (14/14)
All of the symptoms you/your child experience from the mitochondrial disease	93.1 (27/29)	86.7 (13/15)	100.0 (14/14)

[1]Nonrespondents on individual questions (maximum 1 [3.3%] for all, 0 [0.0%] for Adults and 1 [6.7%] for Children) are excluded.

ii. How likely would you/your child be to participate in a clinical trial if it is:

	All N=30	Adults N=15	Children N=15
One day long	89.7 (26/29)	86.7 (13/15)	92.9 (13/14)
One week long	82.8 (24/29)	73.3 (11/15)	92.9 (13/14)
One month long	85.7 (24/28)	78.6 (11/14)	92.9 (13/14)
Several months (3-4 months) in length	78.6 (22/28)	78.6 (11/14)	78.6 (11/14)
One year in length	60.7 (17/28)	57.1 (8/14)	64.3 (9/14)
More than one year in length	53.6 (15/28)	50.0 (7/14)	57.1 (8/14)

[1]Nonrespondents on individual questions (maximum 2 [6.7%] for all, 1 [6.7%] for Adults and 1 [6.7%] for Children) are excluded.

C. Trial Design

i. How likely would you/your child be to participate in a clinical trial if it is:

	All N=30	Adults N=15	Children N=15
Half of the people in the study get a placebo pill (inactive drug) and the other half get the active drug	37.0 (10/27)	46.2 (6/13)	28.6 (4/14)
Half of the people in the study get the active drug and the other half get a placebo pill (inactive drug)	37.0 (10/27)	46.2 (6/13)	28.6 (4/14)
You sequentially take several different drugs or placebos each for a defined time period in an	48.1 (13/27)	61.5 (8/13)	35.7 (5/14)

unpredictable order (ie. Take drug A for one month, then take drug B for one month, then take drug C for one month)			
There is a chance of only getting the placebo (inactive drug)	33.3 (9/27)	38.5 (5/13)	28.6 (4/14)
Everyone gets the drug and placebo at some point	66.7 (18/27)	84.6 (11/13)	50.0 (7/14)
Everyone gets only the drug at some point	77.8 (21/27)	84.6 (11/13)	71.4 (10/14)
Neither you nor the study team know whether you are receiving the drug or placebo	29.6 (8/27)	30.8 (4/13)	28.6 (4/14)
Only you do not know which treatment you are receiving	33.3 (9/27)	38.5 (5/13)	28.6 (4/14)
Only your doctor does not know which treatment you are receiving	18.5 (5/27)	15.4 (2/13)	21.4 (3/14)
The study team selects whether you receive the drug or placebo	33.3 (9/27)	30.8 (4/13)	35.7 (5/14)
You select whether you receive the drug or placebo	51.9 (14/27)	61.5 (8/13)	42.9 (6/14)
There is random assignment of who receives the drug or placebo	37.0 (10/27)	38.5 (5/13)	35.7 (5/14)
You could be randomized to either take the new treatment or continue your regular mitochondrial cocktail	44.4 (12/27)	46.2 (6/13)	42.9 (6/14)
You are already enrolled in another clinical trial at the same time	25.9 (7/27)	30.8 (4/13)	21.4 (3/14)
You are guaranteed the drug after the study ends	55.6 (15/27)	61.5 (8/13)	50.0 (7/14)
[1]Nonrespondents on individual questions (maximum 3 [30.0%] for all, 2 [13.3%] for Adults and 1 [6.7%] for Children) are excluded.			
**ii. How likely would you/your child be to participate if the clinical trial involves:**			
	All N=30	Adults N=15	Children N=15
Daily blood tests	22.2 (6/27)	23.1 (3/13)	21.4 (3/14)
Weekly blood tests	55.6 (15/27)	53.8 (7/13)	57.1 (8/14)
Monthly blood tests	81.5 (22/27)	84.6 (11/13)	78.6 (11/14)
2 blood tests (one at the beginning and one at the end)	76.9 (20/26)	75.0 (9/12)	78.6 (11/14)
Urine tests	80.8 (21/26)	76.9 (10/13)	84.6 (11/13)
Stool tests	70.4 (19/27)	53.8 (7/13)	85.7 (12/14)
An electrocardiogram (ECG)	81.5 (22/27)	76.9 (10/13)	85.7 (12/14)
And echocardiogram (heart ultrasound)	85.2 (23/27)	84.6 (11/13)	85.7 (12/14)
Heart rate monitoring	77.8 (21/27)	76.9 (10/13)	78.6 (11/14)
Exercise tests	66.7 (18/27)	69.2 (9/13)	64.3 (9/14)
An X-ray	77.8 (21/27)	84.6 (11/13)	71.4 (10/14)
An MRI	55.6 (15/27)	69.2 (9/13)	42.9 (6/14)
An ultrasound	81.5 (22/27)	84.6 (11/13)	78.6 (11/14)
Having an IV placed	48.1 (13/27)	30.8 (4/13)	64.3 (9/14)
Visits to the research site or a hospital	70.4 (19/27)	69.2 (9/13)	71.4 (10/14)
Overnight hospital visits	55.6 (15/27)	46.2 (6/13)	64.3 (9/14)
No travelling at all	81.5 (22/27)	76.9 (10/13)	85.7 (12/14)
Travel within a city	88.9 (24/27)	92.3 (12/13)	85.7 (12/14)
Traveling to another state	55.6 (15/27)	38.5 (5/13)	71.4 (10/14)
International travel to another country	25.9 (7/27)	15.4 (2/13)	35.7 (5/14)

Traveling while you are experiencing symptoms	40.7 (11/27)	46.2 (6/13)	35.7 (5/14)
Traveling when you are feeling good enough to travel	63.0 (17/27)	53.8 (7/13)	71.4 (10/14)
No payment or monetary reimbursement	48.1 (13/27)	38.5 (5/13)	57.1 (8/14)
A cash incentive to participate	55.6 (15/27)	61.5 (8/13)	50.0 (7/14)
A gift card incentive to participate	55.6 (15/27)	61.5 (8/13)	50.0 (7/14)
You having to make a payment in order to be part of the trial	18.5 (5/27)	7.7 (1/13)	28.6 (4/14)
[1]Nonrespondents on individual questions (maximum 4 [13.3%] for all, 3 [20.0%] for Adults and 2 [13.3%] for Children) are excluded.			
**iii. How likely would you/your child be to participate if the clinical trial is:**			
	All<br>N=30	Adults<br>N=15	Children<br>N=15
Conducted by your local doctor	85.2 (23/27)	92.3 (12/13)	78.6 (11/14)
Conducted by an academic hospital	88.9 (24/27)	84.6 (11/13)	92.9 (13/14)
Conducted by a pharmaceutical company	40.7 (11/27)	30.8 (4/13)	50.0 (7/14)
Conducted by a patient advocacy group or support group	69.2 (18/26)	53.8 (7/13)	84.6 (11/13)
A single-site trial	66.7 (18/27)	61.5 (8/13)	71.4 (10/14)
A multi-site trial (different locations are working together on the same trial)	66.7 (18/27)	53.8 (7/13)	78.6 (11/14)
In phase 1 (screening for safety)	51.9 (14/27)	53.8 (7/13)	50.0 (7/14)
In phase 2 (establishing the efficacy of the drug, usually against a placebo)	59.3 (16/27)	53.8 (7/13)	64.3 (9/14)
In phase 3 (final confirmation of safety and efficacy)	70.4 (19/27)	61.5 (8/13)	78.6 (11/14)
[1]Nonrespondents on individual questions (maximum 4 [13.3%] for all, 2 [13.3%] for Adults and 2 [13.3%] for Children) are excluded.			
**D. Other Features**			
**i. Would the following factor(s) influence your decision to participate in a clinical trial:**			
	All<br>N=30	Adults<br>N=15	Children<br>N=15
Potential to benefit yourself	84.0 (21/25)	100.0 (12/12)	69.2 (9/13)
Potential to benefit your family	84.0 (21/25)	91.7 (11/12)	76.9 (10/13)
Potential to benefit other affected individuals	80.0 (20/25)	83.3 (10/12)	76.9 (10/13)
Potential to aid in science and scientific advancement	68.0 (17/25)	75.0 (9/12)	61.5 (8/13)
No other treatment options exist	84.0 (21/25)	91.7 (11/12)	76.9 (10/13)
No other affordable treatment options	84.0 (21/25)	83.3 (10/12)	84.6 (11/13)
The same treatment is not available clinically	80.0 (20/25)	75.0 (9/12)	84.6 (11/13)
The same treatment is available outside of the trial but too expensive to access	68.0 (17/25)	58.3 (7/12)	76.9 (10/13)
Access to free healthcare	44.0 (11/25)	41.7 (5/12)	46.2 (6/13)
Apparent risks will outweigh the benefit	0.0 (0/23)	0.0 (0/12)	0.0 (0/11)
Apparent benefits will outweigh the risks	84.0 (21/25)	83.3 (10/12)	84.6 (11/13)
No prospective self benefit	4.2 (1/24)	8.3 (1/12)	0.0 (0/12)
Possibility to cure your disease	96.0 (24/25)	100 (12/12)	92.3 (12/13)
Possibility to prevent progression of your disease	96.0 (24/25)	100 (12/12)	92.3 (12/13)

Possibility to treat some symptoms of your disease	96.0 (24/25)	100 (12/12)	92.3 (12/13)
Potential of worsening your disease	8.3 (2/24)	8.3 (1/12)	8.3 (1/12)
Potential of experiencing transient major side effects	8.3 (2/24)	0.0 (0/12)	16.7 (2/12)
Potential of experiencing transient minor side effects	36.0 (9/25)	33.3 (4/12)	38.5 (5/13)
Potential for death from study participation	0.0 (0/24)	0.0 (0/12)	0.0 (0/12)
Potential for closer monitoring of your health	68.0 (17/25)	66.7 (8/12)	69.2 (9/13)
Potential out-of-pocket expenses	8.3 (2/24)	0.0 (0/12)	16.7 (2/12)
Desire to participate in any clinical trial	27.3 (6/22)	27.3 (3/11)	27.3 (3/11)
Desire to avoid participation in any clinical trial	0.0 (0/22)	0.0 (0/11)	0.0 (0/11)
[1]Nonrespondents on individual questions (maximum 8 [26.7%] for all, 4 [26.7%] for Adults and 4 [26.7%] for Children) are excluded.			
**ii. How likely would you/your child be to participate in a clinical trial if you learned about the trial through:**			
	All N=30	Adults N=15	Children N=15
Your primary care physician	76.0 (19/25)	66.7 (8/12)	84.6 (11/13)
One of your medical specialists	88.0 (22/25)	83.3 (10/12)	92.3 (12/13)
A healthy family member	48.0 (12/25)	41.7 (5/12)	53.8 (7/13)
A family member that was already in the clinical trial	64.0 (16/25)	41.7 (5/12)	84.6 (11/13)
A healthy friend	40.0 (10/25)	33.3 (4/12)	46.2 (6/13)
A friend who also has a mitochondrial disease	80.0 (20/25)	75.0 (9/12)	84.6 (11/13)
Another participant that was already in the clinical trial	84.0 (21/25)	75.0 (9/12)	92.3 (12/13)
A support group or patient advocacy group	76.0 (19/25)	66.7 (8/12)	84.6 (11/13)
The NIH clinical trials website	75.0 (18/24)	63.6 (7/11)	84.6 (11/13)
A newspaper article	40.0 (10/25)	25.0 (3/12)	53.8 (7/13)
A social media website	28.0 (7/25)	16.7 (2/12)	38.5 (5/13)
The internet	28.0 (7/25)	16.7 (2/12)	38.5 (5/13)
The television	28.0 (7/25)	16.7 (2/12)	38.5 (5/13)
A flyer	28.0 (7/25)	16.7 (2/12)	38.5 (5/13)
A letter mailed to your home	56.0 (14/25)	50.0 (6/12)	61.5 (8/13)
A phone call from the study team	80.0 (20/25)	75.0 (9/12)	84.6 (11/13)
An email from the study team	68.0 (17/25)	66.7 (8/12)	69.2 (9/13)
An email from the North American Mitochondrial Disease Consortium (NAMDC)	80.0 (20/25)	66.7 (8/12)	92.3 (12/13)
[1]Nonrespondents on individual questions (maximum 6 [20.0%] for all, 4 [26.7%] for Adults and 2 [13.3%] for Children) are excluded.			
**iii. How likely are you/your child to participate in a clinical trial knowing that:**			
	All N=30	Adults N=15	Children N=15
Your genetic information is being analyzed	80.0 (20/25)	66.7 (8/12)	92.3 (12/13)
Your genetic information cannot affect your medical insurance policy	92.0 (23/25)	83.3 (10/12)	100.0 (13/13)
Your genetic information can affect your ability to purchase a life insurance policy	28.0 (7/25)	8.3 (1/12)	46.2 (6/13)

Your genetic information can affect your ability to qualify for disability insurance	28.0 (7/25)	8.3 (1/12)	46.2 (6/13)
[1]Nonrespondents on individual questions (maximum 5 [16.7%] for All, 3 [10.0%] for Adults and 2 [13.3%] for Children) are excluded.			
