# Supplementary material for: Mitochondrial disease patient motivations and barriers to participate in clinical trials
Source: PLoS One. 2018 May 17;13(5):e0197513. doi: 10.1371/journal.pone.0197513 (PMC5957366; doi:10.1371/journal.pone.0197513)
Supplement: S5 Table — 1Nonrespondents on individual symptoms (maximum n [33, 19.5%] for Adults and n [20, 16.5%] for Children) are excluded. (PDF) [file pone.0197513.s006.pdf]

S5 Table. RDCRN Survey (n=290) respondents likely to participate in clinical trials with differing design and other features.

Questions		% (n)		
**A. Drug Therapy**				
**i. How likely would you/your child be to participate in a clinical trial if it involves:**				
		All<br>N=290	Adults<br>N=169	Children<br>N=121
Taking a new drug that has never been used before on people		47.3 (130/275)	52.5 (84/160)	40.0 (46/115)
Taking a drug that has been used for other purposes, but not for mitochondrial disease		73.8 (203/275)	76.1 (121/159)	70.7 (82/116)
Taking a vitamin		95.6 (263/275)	98.1 (156/159)	92.2 (107/116)
Taking an antioxidant		92.8 (256/276)	96.3 (154/160)	87.9 (102/116)
Taking a natural supplement that is available at health food stores (ie. GNC)		91.3 (252/276)	96.3 (154/160)	84.5 (98/116)
Taking a plant-derived product		87.3 (240/275)	91.9 (147/160)	80.9 (93/115)
Taking a food product		88.8 (245/276)	93.8 (150/160)	81.9 (95/116)
Participating in an exercise test		80.7 (221/274)	85.5 (136/159)	73.9 (85/115)
Making no changes to your current medication		73.2 (199/272)	74.5 (117/157)	71.3 (82/115)
Stopping one of your current medications		48.7 (132/271)	56.1 (88/157)	38.6 (44/114)
Stopping all of your current medications		26.8 (73/272)	33.5 (53/158)	17.5 (20/114)
Changing your diet		77.5 (213/275)	86.8 (138/159)	64.7 (75/116)
[1]Nonrespondents on individual questions (maximum 19 [6.6%] for all, 12 [7.1%] for Adults and 7 [5.8%] for Children) are excluded.				
**ii. How likely would you/your child be to participate in a clinical trial if the drug:**				
		All<br>N=290	Adults<br>N=169	Children<br>N=121
Is a pill		82.6 (228/276)	89.4 (143/160)	73.3 (85/116)
Is an injection		65.8 (181/275)	74.8 (119/159)	53.4 (62/116)
Has to be taken one time a day		91.9 (250/272)	92.4 (145/157)	91.3 (105/115)
Has to be taken two times a day		88.4 (243/275)	88.7 (141/159)	87.9 (102/116)
Has to be taken three times a day		78.2 (215/275)	76.1 (121/159)	81.0 (94/116)
Has to be taken four or more times a day		66.1 (181/274)	66.5 (105/158)	65.5 (76/116)
Can be self administered		87.5 (239/273)	89.2 (140/157)	85.3 (99/116)
Has to be given by a nurse		57.2 (158/276)	57.5 (92/160)	56.9 (66/116)
Has to be given at the hospital		50.0 (135/270)	51.0 (79/155)	48.7 (56/115)
[1]Nonrespondents on individual questions (maximum 20 [6.9%] for all, 14 [8.3%] for Adults and 6 [5.0%] for Children) are excluded.				
**iii. If you/your child were enrolled in a clinical trial:**				
		All<br>N=290	Adults<br>N=169	Children<br>N=121
And the same drug became widely available to all people would you…	Quit the clinical trial	4.8 (13/271)	3.8 (6/157)	6.1 (7/114)
	Stay in the clinical trial	95.2 (258/271)	96.2 (151/157)	93.9 (107/114)
And a comparable drug became widely	Quit the clinical trial	4.1 (11/269)	4.5 (7/156)	3.5 (4/113)

available to all people, would you…	Stay in the clinical trial	95.9 (258/269)	95.5 (149/156)	96.5 (109/113)
And a promising new but completely unrelated drug became widely available to all people would you…	Quit the clinical trial	8.4 (22/263)	9.9 (15/152)	6.3 (7/111)
	Stay in the clinical trial	91.6 (241/263)	90.1 (137/152)	93.7 (104/111)
And you had progression of your disease symptoms while enrolled, would you…	Quit the clinical trial	49.6 (133/268)	46.8 (73/156)	53.6 (60/112)
	Stay in the clinical trial	50.4 (135/268)	53.2 (83/156)	46.4 (52/112)
[1]Nonrespondents on individual questions (maximum 27 [9.3%] for all, 17 [10.1%] for Adults and 10 [8.3%] for Children) are excluded.				

B. Goal of the Study

i. How likely would you/your child be to participate in a clinical trial if it is supposed to help with:

	All N=290	Adults N=169	Children N=121
Only one of the symptoms that you/your child experiences from the mitochondrial disease	86.2 (232/269)	88.4 (137/155)	83.3 (95/114)
Multiple symptoms that you/your child experience but not all of the symptoms	94.4 (253/268)	97.4 (150/154)	90.4 (103/114)
All of the symptoms you/your child experience from the mitochondrial disease	95.9 (257/268)	98.1 (151/154)	93.0 (106/114)
[1]Nonrespondents on individual questions (maximum 22 [7.6%] for all, 15 [8.9%] for Adults and 7 [5.8%] for Children) are excluded.			

ii. How likely would you/your child be to participate in a clinical trial if it is...

	All N=290	Adults N=169	Children N=121
One day long	85.7 (227/265)	85.8 (133/155)	85.5 (94/110)
One week long	86.5 (230/266)	85.8 (133/155)	87.4 (97/111)
One month long	85.0 (226/266)	85.2 (132/155)	84.7 (94/111)
Several months (3-4 months) in length	83.2 (218/262)	84.9 (129/152)	80.9 (89/110)
One year in length	75.4 (199/264)	75.2 (115/153)	75.7 (84/111)
More than one year in length	68.1 (179/263)	66.4 (101/152)	70.3 (78/111)
[1]Nonrespondents on individual questions (maximum 28 [9.7%] for all, 17 [10.1%] for Adults and 11 [9.1%] for Children) are excluded.			

C. Trial Design

i. How likely would you/your child be to participate in a clinical trial if it is..

	All N=290	Adults N=169	Children N=121
Half of the people in the study get a placebo pill (inactive drug) and the other half get the active drug	58.0 (153/264)	59.9 (91/152)	55.4 (62/112)
Half of the people in the study get the active drug and the other half get a placebo pill (inactive drug)	57.0 (150/263)	59.2 (90/152)	54.1 (60/111)

You sequentially take several different drugs or placebos each for a defined time period in an unpredictable order (ie. Take drug A for one month, then take drug B for one month, then take drug C for one month)	58.7 (155/264)	61.8 (94/152)	54.5 (61/112)
There is a chance of only getting the placebo (inactive drug)	46.6 (122/262)	49.0 (74/151)	43.2 (48/111)
Everyone gets the drug and placebo at some point	70.5 (184/261)	70.2 (106/151)	70.9 (78/110)
Everyone gets only the drug at some point	82.0 (214/261)	83.2 (124/149)	80.4 (90/112)
Neither you nor the study team know whether you are receiving the drug or placebo	52.3 (136/260)	53.7 (80/149)	50.5 (56/111)
Only you do not know which treatment you are receiving	53.8 (141/262)	54.7 (82/150)	52.7 (59/112)
Only your doctor does not know which treatment you are receiving	51.1 (134/262)	53.0 (80/151)	48.6 (54/111)
The study team selects whether you receive the drug or placebo	51.9 (136/262)	53.3 (80/150)	50.0 (56/112)
You select whether you receive the drug or placebo	64.8 (169/261)	67.8 (101/149)	60.7 (68/112)
There is random assignment of who receives the drug or placebo	60.5 (158/261)	61.3 (92/150)	59.5 (66/111)
You could be randomized to either take the new treatment or continue your regular mitochondrial cocktail	66.3 (173/261)	65.1 (97/149)	67.9 (76/112)
You are already enrolled in another clinical trial at the same time	38.5 (100/260)	36.2 (54/149)	41.4 (46/111)
You are guaranteed the drug after the study ends	80.6 (208/258)	81.6 (120/147)	79.3 (88/111)
[1]Nonrespondents on individual questions (maximum 32 [11.0%] for all, 22 [13.0%] for Adults and 11 [9.1%] for Children) are excluded.			
**ii. How likely would you/your child be to participate if the clinical trial involves...**			
	All N=290	Adults N=169	Children N=121
Daily blood tests	35.6 (93/261)	40.1 (61/152)	29.4 (32/109)
Weekly blood tests	63.4 (166/262)	69.1 (105/152)	55.5 (61/110)
Monthly blood tests	81.2 (211/260)	86.1 (130/151)	74.3 (81/109)
2 blood tests (one at the beginning and one at the end)	85.6 (225/263)	86.2 (131/152)	84.7 (94/111)
Urine tests	84.8 (223/263)	86.8 (132/152)	82.0 (91/111)
Stool tests	74.8 (196/262)	72.4 (110/152)	78.2 (86/110)
An electrocardiogram (ECG)	84.8 (223/263)	85.5 (130/152)	83.8 (93/111)
And echocardiogram (heart ultrasound)	85.2 (224/263)	86.2 (131/152)	83.8 (93/111)
Heart rate monitoring	84.3 (220/261)	84.8 (128/151)	83.6 (92/110)
Exercise tests	78.6 (206/262)	82.2 (125/152)	73.6 (81/110)
An X-ray	78.9 (206/261)	79.5 (120/151)	78.2 (86/110)
An MRI	76.3 (200/262)	77.6 (118/152)	74.5 (82/110)
An ultrasound	83.6 (219/262)	85.5 (130/152)	80.9 (89/110)
Having an IV placed	64.6 (166/257)	68.5 (102/149)	59.3 (64/108)
Visits to the research site or a hospital	72.1 (189/262)	74.3 (113/152)	69.1 (76/110)
Overnight hospital visits	66.7 (174/261)	70.2 (106/151)	61.8 (68/110)
No travelling at all	82.1 (215/262)	79.6 (121/152)	85.5 (94/110)

Traveling within same city or town	83.5 (218/261)	80.8 (122/151)	87.3 (96/110)
Traveling to another state	60.9 (156/256)	60.4 (90/149)	61.7 (66/107)
International travel to another country	39.7 (104/262)	38.8 (59/152)	40.9 (45/110)
Traveling while you are experiencing symptoms	55.2 (143/259)	59.2 (90/152)	49.5 (53/107)
Traveling when you are feeling good enough to travel	70.0 (182/260)	72.7 (109/150)	66.4 (73/110)
No payment or monetary reimbursement	60.9 (159/261)	61.2 (93/152)	60.6 (66/109)
A cash incentive to participate	69.7 (182/261)	72.4 (110/152)	66.1 (72/109)
A gift card incentive to participate	67.4 (174/258)	70.0 (105/150)	63.9 (69/108)
You having to make a payment in order to be part of the trial	18.8 (49/260)	16.7 (25/150)	21.8 (24/110)
[1]Nonrespondents on individual questions (maximum 33 [11.4%] for all, 20 [11.8%] for Adults and 14 [11.6%] for Children) are excluded.			
**iii. How likely would you/your child be to participate if the clinical trial is:**			
	All N=290	Adults N=169	Children N=121
Conducted by your local doctor	87.8 (230/262)	89.5 (136/152)	85.5 (94/110)
Conducted by an academic hospital	86.2 (225/261)	88.7 (134/151)	82.7 (91/110)
Conducted by a pharmaceutical company	64.2 (167/260)	65.6 (99/151)	62.4 (68/109)
Conducted by a patient advocacy group or support group	70.3 (182/259)	71.8 (107/149)	68.2 (75/110)
A single-site trial	70.7 (181/256)	71 (105/147)	69.7 (76/109)
A multi-site trial (different locations are working together on the same trial)	74.2 (193/260)	76.5 (114/149)	71.2 (79/111)
In phase 1 (screening for safety)	58.9 (152/258)	64.9 (96/148)	50.9 (56/110)
In phase 2 (establishing the efficacy of the drug, usually against a placebo)	74.7 (195/261)	75.3 (113/150)	73.9 (82/111)
In phase 3 (final confirmation of safety and efficacy)	81.2 (212/261)	80.7 (121/150)	82.0 (91/111)
1Nonrespondents on individual questions (maximum 34 [11.7%] for all, 22 [13.0%] for Adults and 12 [9.9%] for Children) are excluded.			
**D. Other Features**			
**i. Would the following factor(s) influence your decision to participate in a clinical trial:**			
	All N=290	Adults N=169	Children N=121
Potential to benefit yourself	83.3 (219/263)	89.5 (137/153)	74.5 (82/110)
Potential to benefit your family	83.8 (217/259)	85.4 (129/151)	81.5 (88/108)
Potential to benefit other affected individuals	83.6 (219/262)	87.4 (132/151)	78.4 (87/111)
Potential to aid in science	74.2 (193/260)	78.8 (119/151)	67.9 (74/109)
No other treatment options exist	78.8 (205/260)	75.5 (114/151)	83.5 (91/109)
No other affordable treatment options	75.4 (196/260)	72.2 (109/151)	79.8 (87/109)
The same treatment is not available clinically	74.1 (192/259)	69.5 (105/151)	80.6 (87/108)
The same treatment is available outside of the trial but too expensive to access	70.0 (182/260)	68.4 (104/152)	72.2 (78/108)
Access to free healthcare	61.3 (160//261)	60.5 (92/152)	62.4 (68/109)
Apparent risks will outweigh the benefit	23.4 (60/256)	25.3 (38/150)	20.8 (22/106)
No prospective self-benefit	19.8 (51/257)	24.7 (37/150)	13.1 (14/107)
Possibility to cure your disease	91.6 (239/261)	90.7 (137/151)	92.7 (102/110)

Possibility to prevent progression of your disease	92.7 (240/259)	92.0 (138/150)	93.6 (102/109)
Possibility to treat some symptoms of your disease	91.1 (235/258)	91.3 (137/150)	90.7 (98/108)
Potential of worsening your disease	10.5 (27/257)	12.7 (19/150)	7.5 (8/107)
Potential of experiencing transient major side effects	10.6 (27/255)	12.7 (19/150)	7.6 (8/105)
Potential of experiencing transient minor side effects	34.0 (87/256)	33.8 (51/151)	34.3 (36/105)
Potential for death from study participation	7.0 (18/257)	8.7 (13/150)	4.7 (5/107)
Potential for closer monitoring of your health	67.7 (174/257)	67.3 (101/150)	68.2 (73/107)
Potential out-of-pocket expenses	13.3 (34/255)	12.9 (19/147)	13.9 (15/108)
Desire to participate in any clinical trial	42.7 (109/255)	46.3 (69/149)	37.7 (40/106)
Desire to avoid participation in any clinical trial	9.3 (23/247)	12.6 (18/143)	4.8 (5/104)
[1]Nonrespondents on individual questions (maximum 34 [11.7%] for all, 26 [15.4%] for Adults and 17 [14.0%] for Children) are excluded.			
**ii. How likely would you/your child be to participate in a clinical trial if you learned about the trial through:**			
	All N=290	Adults N=169	Children N=121
Your primary care physician	79.7 (208/261)	81.6 (124/152)	77.1 (84/109)
One of your medical specialists	89.7 (234/261)	90.8 (138/152)	88.1 (96/109)
A healthy family member	54.8 (143/261)	58.6 (89/152)	49.5 (54/109)
A family member that was already in the clinical trial	66.9 (174/260)	66.4 (101/152)	67.6 (73/108)
A healthy friend	49.0 (128/261)	53.3 (81/152)	43.1 (47/109)
A friend who also has a mitochondrial disease	80.7 (209/259)	82.9 (126/152)	77.6 (83/107)
Another participant that was already in the clinical trial	78.5 (204/260)	78.3 (119/152)	78.7 (85/108)
A support group or patient advocacy group	75.2 (194/258)	75.3 (113/150)	75.0 (81/108)
The NIH clinical trials website	78.6 (202/257)	81.9 (122/149)	74.1 (80/108)
A newspaper article	42.8 (110/257)	43.3 (65/150)	42.1 (45/107)
A social media website	38.4 (99/258)	40.4 (61/151)	35.5 (38/107)
The internet	42.5 (110/259)	45.0 (68/151)	38.9 (42/108)
The television	37.5 (97/259)	39.7 (60/151)	34.3 (37/108)
A flyer	37.5 (96/256)	39.3 (59/150)	34.9 (37/106)
A letter mailed to your home	63.2 (163/258)	64.9 (98/151)	60.7 (65/107)
A phone call from the study team	73.4 (190/259)	75.7 (115/152)	70.1 (75/107)
An email from the study team	74.8 (193/258)	75.7 (115/152)	73.6 (78/106)
An email from the North American Mitochondrial Disease Consortium (NAMDC)	83.8 (217/259)	83.6 (127/152)	84.1 (90/107)
[1]Nonrespondents on individual questions (maximum 34 [11.7%] for all, 20 [11.8%] for Adults and 15 [16.5%] for Children) are excluded.			
**iii. How likely are you/your child to participate in a clinical trial knowing that:**			
	All N=290	Adults N=169	Children N=121
Your genetic information is being analyzed	83.7 (200/239)	84.1 (116/138)	83.2 (84/101)
Your genetic information cannot affect your medical insurance policy	84.8 (201/237)	86.0 (117/136)	83.2 (84/101)

Your genetic information can affect your ability to purchase a life insurance policy	42.0 (100/238)	44.5 (61/137)	38.6 (39/101)
Your genetic information can affect your ability to qualify for disability insurance	36.8 (88/239)	37.7 (51/138)	36.6 (37/101)
[1]Nonrespondents on individual questions (maximum 53 [18.3%] for all, 33 [19.5%] for Adults and 20 [16.5%] for Children) are excluded.			

[1]Nonrespondents on individual symptoms (maximum n [33, 19.5%] for Adults and n [20, 16.5%] for Children) are excluded.
