# Supplementary material for: Mitochondrial disease patient motivations and barriers to participate in clinical trials
Source: PLoS One. 2018 May 17;13(5):e0197513. doi: 10.1371/journal.pone.0197513 (PMC5957366; doi:10.1371/journal.pone.0197513)
Supplement: S6 Table — 1Respondents who had participated in a previous study or clinical trial were coded as having past research experience (N = 98). (PDF) [file pone.0197513.s007.pdf]

S6 Table. Logistic Regression analysis of impact of past research experience[1] on preferences for study drug and design features among Adults and Children combined (N=290).

Study drug or Study Design	Odds Ratio	95% CI	p-value
Drug had not been used before	1.17	0.73-1.88	0.52
Injectable medication	0.84	0.50-1.41	0.50
Stopping one medication	0.97	0.60-1.56	0.90
Stopping all medications	0.99	0.62-1.57	0.96
More than 1 year in length	1.24	0.72-2.17	0.44
Daily blood tests	0.78	0.48-1.27	0.32
Placebo	0.88	0.54-1.42	0.60
Double blinded	1.20	0.74-1.96	0.46
Randomization	0.99	0.61-1.61	0.97

[1] Respondents who had participated in a previous study or clinical trial were coded as having past research experience (N= 98).
