# Supplementary material for: Mitochondrial disease patient motivations and barriers to participate in clinical trials
Source: PLoS One. 2018 May 17;13(5):e0197513. doi: 10.1371/journal.pone.0197513 (PMC5957366; doi:10.1371/journal.pone.0197513)
Supplement: S7 Table — (PDF) [file pone.0197513.s008.pdf]

S7 Table. P-values from the Wilcoxon Rank-Sum Test (n=263) for Severity Score Measurement.

Method	Dichotomize Above/Below Median	Median Severity Score	Group Size		P-value
			Less severe symptoms	More severe symptoms	
1	above	5	147	116	0.1379
1	below	5	120	143	0.1579
