# Supplementary material for: Mitochondrial disease patient motivations and barriers to participate in clinical trials
Source: PLoS One. 2018 May 17;13(5):e0197513. doi: 10.1371/journal.pone.0197513 (PMC5957366; doi:10.1371/journal.pone.0197513)
Supplement: S8 Table — (PDF) [file pone.0197513.s009.pdf]

S8 Table. Summary of likelihood of participation in a clinical trial in all CHOP(N=30) and RDCRN (N=290) subjects.

**Drug Therapy**	**CHOP survey % (n)**	**RDCRN survey % (n)**
Taking a vitamin	96.7 (29/30)	95.6 (263/275)
Taking an antioxidant	90.0 (27/30)	92.8 (256/276)
Taking a natural supplement that is available at health food stores (ie. GNC)	96.7 (29/30)	91.3 (252/276)
Taking a plant-derived product	83.3 (25/30)	87.3 (240/275)
Taking a food product	93.3 (28/30)	88.8 (245/276)
Participating in an exercise test	72.4 (21/29)	80.7 (221/274)
Is a pill	83.3 (25/30)	82.6 (228/276)
Has to be taken one time a day	93.1 (27/29)	91.9 (250/272)
Has to be taken two times a day	90.0 (27/30)	88.4 (243/275)
Can be self administered	69 (20/29)	87.5 (239/273)
The same drug became widely available	93.3 (28/30)	95.2 (258/271)
A comparable drug became widely available	96.6 (28/29)	95.9 (258/269)
A promising new but completely unrelated drug became widely available	82.8 (24/29)	91.6 (241/263)
**Goal of the Study**		
Multiple symptoms that you/your child experience but not all of the symptoms	93.1 (27/29)	94.4 (253/268)
All of the symptoms you/your child experience from the mitochondrial disease	93.1 (27/29)	95.9 (257/268)
One day long	89.7 (26/29)	85.7 (227/265)
One week long	82.8 (24/29)	86.5 (230/266)
One month long	85.7 (24/28)	85.0 (226/266)
Several months (3-4 months) in length	78.6 (22/28)	83.2 (218/262)
**Trial Design**		
Everyone gets only the drug at some point	77.8 (21/27)	82.0 (214//261)
You are guaranteed the drug after the study ends	55.6 (15/27)	80.6 (208/258)
Monthly blood tests	81.5 (22/27)	81.2 (211/260)
2 blood tests (one at the beginning and one at the end)	76.9 (20/26)	85.6 (225/263)
Urine tests	80.8 (21/26)	84.8 (223/263)
An electrocardiogram (ECG)	81.5 (22/27)	84.8 (223/263)
And echocardiogram (heart ultrasound)	85.2 (23/27)	85.2 (224/263)
Heart rate monitoring	77.8 (21/27)	84.3 (220/261)
An ultrasound	81.5 (22/27)	83.6 (219/262)
No travelling at all	81.5 (22/27)	82.1 (215/262)
Traveling within same city or town	88.9 (24/27)	83.5 (218/261)
Conducted by your local doctor	85.2 (23/27)	87.8 (230/262)
Conducted by an academic hospital	88.9 (24/27)	86.2 (225/261)
In phase 3 (final confirmation of safety and efficacy)	-	81.2 (212/261)
**Other Features**		
Potential to benefit yourself	84.0 (21/25)	83.3 (219/263)
Potential to benefit your family	84.0 (21/25)	83.8 (217/259)
Potential to benefit other affected individuals	80.0 (20/25)	83.6 (219/262)
No other treatment options exist	84.0 (21/25)	78.8 (205/260)
No other affordable treatment options	84.0 (21/25)	75.4 (196/260)
The same treatment is not available clinically	80.0 (20/25)	74.1 (192/259)

Possibility to cure your disease	96.0 (24/25)	91.6 (239/261)
Possibility to prevent progression of your disease	96.0 (24/25)	92.7 (240/259)
Possibility to treat some symptoms of your disease	94.0 (24/25)	91.1 (235/258)
**How likely would you/your child be to participate in a clinical trial if you learned about the trial through:**		
One of your medical specialists	88.0 (22/25)	89.7 (234/261)
A friend who also has a mitochondrial disease	80.0 (20/25)	80.7 (209/259)
Another participant that was already in the clinical trial	84.0 (21/25)	
A phone call from the study team	80.0 (20/25)	
An email from the North American Mitochondrial Disease Consortium (NAMDC)	80.0 (20/25)	83.8 (217/259)
Your genetic information is being analyzed	80.0 (20/25)	83.7 (200/239)
Your genetic information cannot affect your medical insurance policy	92.0 (23/25)	84.8 (201/237)
