# Supplementary material for: Mitochondrial disease patient motivations and barriers to participate in clinical trials
Source: PLoS One. 2018 May 17;13(5):e0197513. doi: 10.1371/journal.pone.0197513 (PMC5957366; doi:10.1371/journal.pone.0197513)
Supplement: S9 Table — (PDF) [file pone.0197513.s010.pdf]

S9 Table. Summary of discouraging factors in a clinical trial among all CHOP (N=30) and RDCRN (N=290) subjects.

**Drug Therapy**	**CHOP survey % (n)**	**RDCRN survey % (n)**
Taking a new drug that has never been used before on people	20.0 (6/30)	47.3 (130/275)
Taking a drug that has been used for other purposes, but not for mitochondrial disease	70.0 (21/30)	73.8 (203/275)
Making no changes to your current medication	66.7 (20/30)	73.2 (199/272)
Stopping one of your current medications	50.0 (15/30)	48.7 (132/271)
Stopping all of your current medications	20.0 (6/30)	26.8 (73/272)
Changing your diet	70.0 (21/30)	77.5 (213/275)
Is an injection	46.7 (14/30)	65.8 (181/275)
Has to be taken three times a day	73.3 (22/30)	78.2 (215/275)
Has to be taken four or more times a day	63.3 (19/30)	66.1 (181/274)
Has to be given by a nurse	43.3 (13/30)	57.2 (158/276)
Has to be given at the hospital	30.0 (9/30)	50.0 (135/270)
Progression of your disease symptoms while enrolled	46.7 (14/30)	50.4 (135/268)
**Goal of the Study**		
More than one year in length	53.6 (15/28)	75.4 (199/264)
**Trial Design**		
Half of the people in the study get a placebo pill (inactive drug) and the other half get the active drug	37.0 (10/27)	58.0 (153/264)
Half of the people in the study get the active drug and the other half get a placebo pill (inactive drug)	37.0 (10/27)	57.0 (150/263)
You sequentially take several different drugs or placebos each for a defined time period in an unpredictable order (ie. Take drug A for one month, then take drug B for one month, then take drug C for one month)	48.1 (13/27)	58.7 (155/264)
There is a chance of only getting the placebo (inactive drug)	33.3 (9/27)	46.6 (122/262)
Everyone gets the drug and placebo at some point	66.7 (18/27)	70.5 (184/261)
Neither you nor the study team know whether you are receiving the drug or placebo	29.6 (8/27)	52.3 (136/260)
Only you do not know which treatment you are receiving	33.3 (9/27)	53.8 (141/262)
Only your doctor does not know which treatment you are receiving	18.5 (5/27)	51.1 (134/262)
The study team selects whether you receive the drug or placebo	33.3 (9/27)	51.9 (136/262)
You select whether you receive the drug or placebo	51.9 (14/27)	64.8 (169/261)
There is random assignment of who receives the drug or placebo	37.0 (10/27)	60.5 (158/261)
You could be randomized to either take the new treatment or continue your regular mitochondrial cocktail	44.4 (12/27)	66.3 (173/261)

You are already enrolled in another clinical trial at the same time	25.9 (7/27)	38.5 (100/260)
Daily blood tests	22.2 (6/27)	35.6 (93/261)
Weekly blood tests	55.6 (15/27)	63.4 (166/262)
Stool tests	70.4 (19/27)	74.8 (196/262)
Having an IV placed	48.1 (13/27)	64.6 (166/257)
Visits to the research site or a hospital	70.4 (19/27)	72.1 (189/262)
Overnight hospital visits	55.6 (15/27)	66.7 (174/261)
Traveling to another state	55.6 (15/27)	60.9 (156/256)
International travel to another country	25.9 (7/27)	39.7 (104/262)
Traveling while you are experiencing symptoms	40.7 (11/27)	55.2 (143/259)
Traveling when you are feeling good enough to travel	63.0 (17/27)	70.0 (182/260)
No payment or monetary reimbursement	48.1 (13/27)	60.9 (159/261)
A cash incentive to participate	55.6 (15/27)	69.7 (182/261)
A gift card incentive to participate	55.6 (15/27)	67.4 (174/258)
You having to make a payment in order to be part of the trial	18.5 (5/27)	18.8 (49/260)
Conducted by a pharmaceutical company	40.7 (11/27)	64.2 (167/260)
Conducted by a patient advocacy group or support group	69.2 (18/26)	70.3 (182/259)
A single-site trial	66.7 (18/27)	70.7 (181/256)
A multi-site trial (different locations are working together on the same trial)	66.7 (18/27)	74.2 (193/260)
In phase 1 (screening for safety)	51.9 (14/27)	58.9 (152/258)
In phase 2 (establishing the efficacy of the drug, usually against a placebo)	59.3 (16/27)	74.7 (195/261)
**Other Features**		
Potential to aid in science and scientific advancement	68.0 (17/25)	74.2 (193/260)
The same treatment is available outside of the trial but too expensive to access	68.0 (17/25)	70.0 (182/260)
Access to free healthcare	44.0 (11/25)	61.3 (160/261)
Apparent risks will outweigh the benefit	0.0 (0/23)	23.4 (60/256)
No prospective self benefit	4.2 (1/24)	19.8 (51/257)
Potential of worsening your disease	8.3 (2/24)	10.5 (27/257)
Potential of experiencing transient major side effects	8.3 (2/24)	10.6 (27/255)
Potential of experiencing transient minor side effects	36.0 (9/25)	34.0 (87/256)
Potential for death from study participation	0.0 (0/24)	7.0 (18/257)
Potential for closer monitoring of your health	68.0 (17/25)	67.7 (174/257)
Potential out-of-pocket expenses	8.3 (2/24)	13.3 (34/255)
Desire to participate in any clinical trial	27.3 (6/22)	42.7 (109/255)
Desire to avoid participation in any clinical trial	0.0 (0/22)	9.3 (23/247)
**How likely would you/your child be to participate in a clinical trial if you learned about the trial through:**		
Your primary care physician	76.0 (19/25)	79.7 (208/261)
A healthy family member	48.0 (12/25)	54.8 (143/261)
A family member that was already in the clinical trial	64.0 (16/25)	66.9 (174/260)
A healthy friend	40.0 (10/25)	49.0 (128/261)

A support group or patient advocacy group	76.0 (19/25)	75.2 (194/258)
The NIH clinical trials website	75.0 (18/24)	78.6 (202/257)
A newspaper article	40.0 (10/25)	42.8 (110/257)
A social media website	28.0 (7/25)	38.4 (99/258)
The internet	28.0 (7/25)	42.5 (110/259)
The television	28.0 (7/25)	37.5 (97/259)
A flyer	28.0 (7/25)	37.5 (96/256)
A letter mailed to your home	56.0 (14/25)	63.2 (163/258)
An email from the study team	68.0 (17/25)	74.8 (193/258)
Your genetic information can affect your ability to purchase a life insurance policy	28.0 (7/25)	42.0 (100/238)
Your genetic information can affect your ability to qualify for disability insurance	28.0 (7/25)	36.8 (88/239)
