# Supplementary figures and images for: Mitochondrial disease patient motivations and barriers to participate in clinical trials
Source: PLoS One. 2018 May 17;13(5):e0197513. doi: 10.1371/journal.pone.0197513 (PMC5957366; doi:10.1371/journal.pone.0197513)

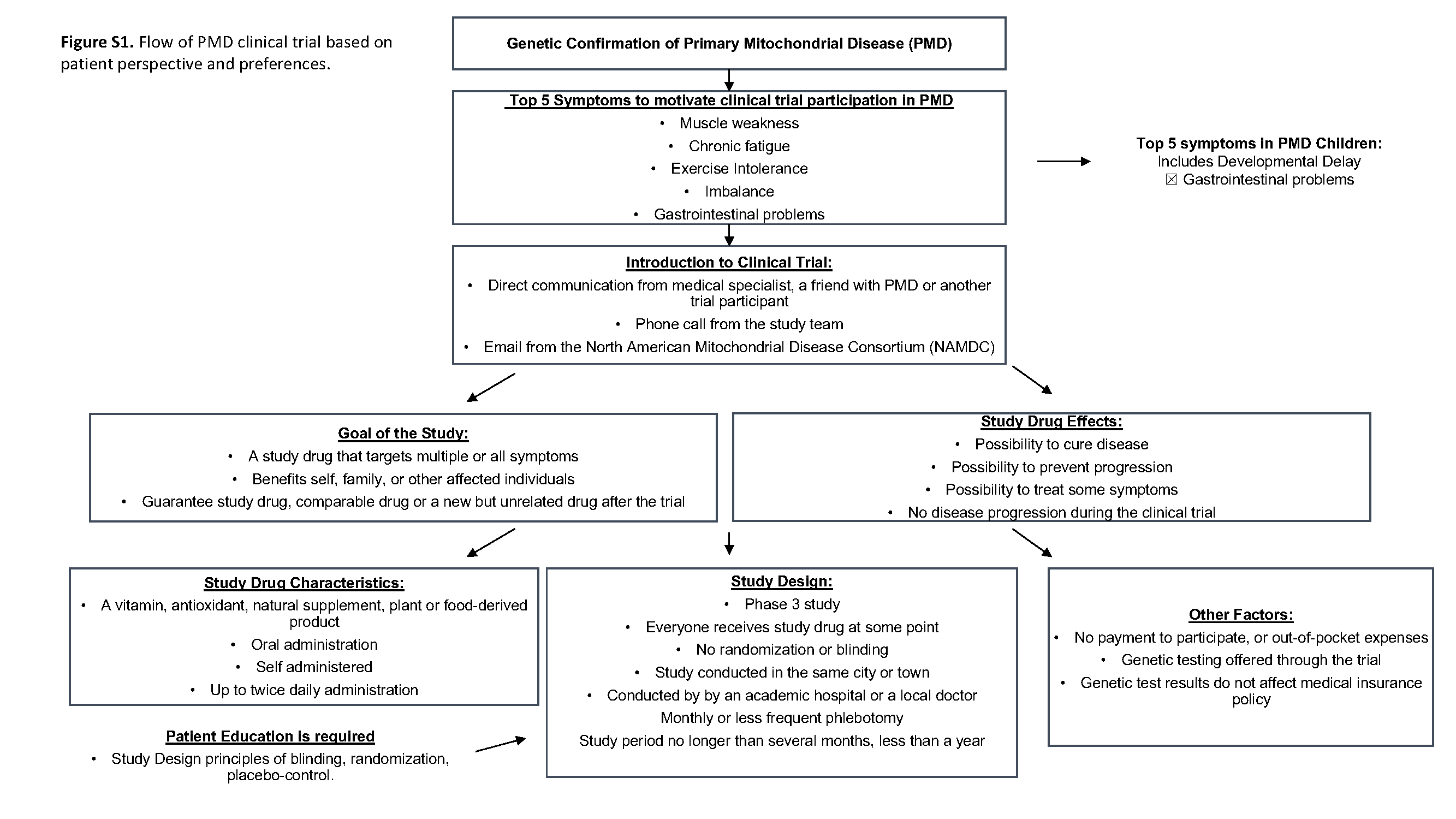

Supplement: S1 Fig — (TIFF) [file pone.0197513.s011.tiff]
